# STRIP2 motivates non-small cell lung cancer progression by modulating the TMBIM6 stability through IGF2BP3 dependent

**DOI:** 10.1186/s13046-022-02573-1

**Published:** 2023-01-13

**Authors:** Xilin Zhang, Qiuqiang Chen, Ying He, Qian Shi, Chengyi Yin, Yanping Xie, Huanming Yu, Ying Bao, Xiang Wang, Chengwu Tang, Zhaohui Dong

**Affiliations:** 1 Huzhou Key Laboratory of Translational Medicine, First Affiliated Hospital of Huzhou University, Huzhou, 313000 Zhejiang China; 2 Department of Cardiothoracic Surgery, First Affiliated Hospital of Huzhou University, Huzhou, 313000 Zhejiang China

**Keywords:** Striatin interacting protein 2, Non-small cell lung cancer, Insulin-like growth factor II mRNA binding protein 3, Transmembrane Bax inhibitor motif containing-6, Metastasis

## Abstract

**Background:**

Striatin interacting protein 2 (STRIP2) is a core component of the striatin-interacting phosphatase and kinase (STRIPAK) complexes, which is involved in tumor initiation and progression via the regulation of cell contractile and metastasis. However, the underlying molecular mechanisms of STRIP2 in non-small cell lung cancer (NSCLC) progression remain largely unknown.

**Methods:**

The expressions of STRIP2 and IGF2BP3 in human NSCLC specimens and NSCLC cell lines were detected using quantitative RT-PCR, western blotting, and immunohistochemistry (IHC) analyses. The roles and molecular mechanisms of STRIP2 in promoting NSCLC progression were investigated in vitro and in vivo.

**Results:**

Here, we found that STRIP2 expression was significantly elevated in NSCLC tissues and high STRIP2 expression was associated with a poor prognosis. Knockdown of STRIP2 suppressed tumor growth and metastasis in vitro and in vivo, while STRIP2 overexpression obtained the opposite effect. Mechanistically, P300/CBP-mediated H3K27 acetylation activation in the promoter of STRIP2 induced STRIP2 transcription, which interacted with insulin-like growth factor 2 mRNA-binding protein 3 (IGF2BP3) and upregulated IGF2BP3 transcription. In addition, STRIP2-IGF2BP3 axis stimulated m6A modification of TMBIM6 mRNA and enhanced TMBIM6 stability. Consequently, TMBIM6 involved NSCLC cell proliferation, migration and invasion dependent on STRIP2 and IGF2BP3. In NSCLC patients, high co-expression of STRIP2, IGF2BP3 and TMBIM6 was associated with poor outcomes.

**Conclusions:**

Our findings indicate that STRIP2 interacts with IGF2BP3 to regulate TMBIM6 mRNA stability in an m6A-dependent manner and may represent a potential prognostic biomarker and therapeutic target for NSCLC.

**Supplementary Information:**

The online version contains supplementary material available at 10.1186/s13046-022-02573-1.

## Background

Lung cancer is the most common malignant tumors globally and is the primary cause of cancer-related death [1, 2]. It mainly contains non-small cell lung cancer (NSCLC) and small cell lung cancer (SCLC). NSCLC consists of 75–85% of all lung cancer patients [1, 2]. About 40–60% patients have advanced stage and metastatic disease at the time of diagnosis [3, 4]. However, surgical resection is generally not suitable for patients with advanced stage. The recommended therapeutic strategy is integrated and systemic therapy (for example, chemotherapy, radiation therapy, molecular targeted therapy, or immunotherapy) [5]. But the five-year survival rate of patients with advanced stage is only 5%, owing to distant metastasis. Previous studies have shown that the most frequent metastatic sites of NSCLC are lung, bone, brain and liver [6, 7]. Therefore, it is helpful to formulate a reasonable postoperative follow-up plan and take targeted intervention measures, with screening metastatic-related proteins and studying the mechanisms of metastasis of NSCLC.

Striatin-interacting phosphatase and kinase (STRIPAK) is a conversed complex, which consists of three parts, including the PP2A scaffolding subunit (A subunit, also known as PP2AA), PP2A catalytic subunit (C subunit, also known as PP2AC), and regulatory subunit striatin (STRN) family proteins containing STRN, STRN3, and STRN4 [8]. Other major components of STRIPAK complexes contain striatin-interacting protein 1 or 2 (STRIP1 or STRIP2, also named as family with sequence similarity 40 (FAM40) A or B), the MOB family member 4 protein (MOB4), the cerebral cavernous malformation 3 protein (CCM3), and the germinal center kinase III (GCKIII) family kinase (MST3, MST4, or STK25) [9, 10]. Recently, STRIPAK complexes show many physiological and pathological functions, and dysregulation of STRIPAK components is participated in cancer [11]. Previous study has shown that STRIPAK components FAM40A, FAM40B, and STRN3 determine the model of cancer cell migration and metastasis through regulating the contractile cytoskeleton phenotype [12]. Kim et al demonstrates that STRIPAK component STRN4 promotes oncogenic transformation of cancer cell via facilitating PP2A-mediated dephosphorylation of MAP 4 K4 [13]. Numerous reports have demonstrated that STRIP1 and STRIP2 are recognized as regulators of cell morphology and cytoskeleton phenotype, [14] which contain two conserved domains, the N-terminal N1221-like domain and the C-terminal DUF3402 (domain of unknown function 3402). The function of both domains is yet to be uncharacterized. Although depletion of STRIP2 does not influence PC3 cell migration, it causes cell elongation and tail retraction defects [14]. In addition, knockdown of STRIP2 induces upregulated transcription of pluripotency factors and epigenetic factors in mouse embryonic stem cells [15]. Recently, Qiu et al has suggested that STRIP2 is highly expressed in lung adenocarcinoma and promoted cell migration via AKT/m-TOR pathway in vitro [16]. In addition, the lncRNA TMPO-AS1/let-7c-5p/STRIP2 axis has acted as a crucial role in lung adenocarcinoma progression and served as an unfavorable survival and tumor infiltration inflation markers [17]. However, the biological roles and molecular mechanisms of STRIP2 in NSCLC are not fully understood.

The present study aimed to reveal the biological roles and molecular mechanisms of STRIP2 in the tumorigenesis of NSCLC and proposed that STRIP2 may be a novel potential prognostic biomarker and therapeutic target for NSCLC progression.

## Materials and methods

### Patient samples

A total of 240 NSCLC and paired para-carcinoma tissues were available from patients who underwent surgical resections at the First Affiliated Hospital of Huzhou University between January, 2015 and December, 2018. Among them, 189 pairs of formalin-fixed paraffin-embedded (FFPE) tissues were consisted of a NSCLC tissue microarray (TMA) and analyzed using immunohistochemistry (IHC) staining, and 51 pairs of fresh tissues were used for reverse transcription-quantitative PCR and western blotting analyses. These patients were not received chemotherapy or radiation therapy superior to surgical resection. This research was approved by the Ethics Committee of the First Affiliated Hospital of Huzhou University (approved number: 2021KYLL007). Informed consents were obtained from all patients. All patients clinicopathological characteristic was shown in Additional files Table S1.

### TCGA datasets

Four hundred seventy-two samples from 1 GEO datasets (GSE32863 [18]), Hou Lung and Okayama Lung [19, 20] were used to analyze the STRIP2 expression in NSCLC compared with normal tissues. In addition, GSE32863 dataset was used to analyze the IGF2BP3, P300 and CBP expressions in NSCLC. Two published IGF2BP3 RIP-sequence data [21] and GEO database (GSE90684) [21] were analyzed.

### Kaplan-Meier plotter

The Kaplan-Meier plotter (www.kmplot.com) [22] was used to predict the relationship between STRIP2 mRNA expression and overall survival as well as recurrence-free survival. STRIP2 expression levels in patients with lung adenocarcinoma (*n* = 601) were collected using Kaplan-Meier Plotter online bioinformatics datasets. Additionally, patients were divided into low or high STRIP2 expression groups, according to STRIP2 mRNA expression based on the median value. The hazard ratio (HR) with 95% confidence intervals (CI) and the log-rank *P* value were calculated in the study.

### Cell culture

Human normal bronchial epithelial cell line (BEAS-2B) and NSCLC cell lines (SPCA1, H1299, A549, PC-9, H1975, H23 and H226) were purchased from the Shanghai Cell Bank of the Chinese Academy of Sciences (Shanghai, China). Cells were cultured in Dulbecco’s Modified Eagle Medium (DMEM, cat. no. L110KJ, BasalMedia, Shanghai) or RPMI-1640 (cat.no. L210KJ, BasalMedia, Shanghai) supplemented with 10% fetal bovine serum (FBS, Gibco, Thermo Fisher Scientific), 100 U/ml penicillin and 100 μg/ml streptomycin (Sigma-Aldrich, Merck KGaA) in a humidified incubator under 5% CO_2_ at 37 °C.

### Plasmid construction and cell transfection

Overexpression plasmids were cloned and constructed into a pcDNA3.1/myc-his (cat.no. V800–20, Invitrogen, Thermo Fisher Scientific, Inc.) or pcDNA3.1-3xFlag (VT8001, Youbio company, Hunan) or pXJ40-Flag (Our laboratory constructed) or pGEX-4 T-1 vector (VT1253, Youbio company, Hunan). For gene knockdown, sh-control (referred to as shCtrl), STRIP2 shRNAs (shSTRIP2#1/2) and IGF2BP3 shRNAs (shIGF2BP3#1/2) were designed and synthesized by Tsingke biological Technology (Hangzhou, China). In addition, two specific small interfering (si) RNAs of P300/CBP/TMBIM6 (20 μM) and scrambled siRNA (referred to as si-Ctrl; 20 μM) were designed and synthesized by Guangzhou RiboBio Co., Ltd. Cells were transfected with Lipofectamine® 2000 reagents (Invitrogen, Thermo Fisher Scientific, Inc.) according to the manufacturer’s instructions. Stable cell lines were selected using puromycin (cat.no. A1113803, Gibco, Thermo Fisher Scientific, Inc.). The primer sequences used in the experiment were as follows: shSTRIP2#1 targeted sequence, 5′-GGAACAAGTTCATCGGATT − 3′; shSTRIP2#2 targeted sequence, 5′-GCCGGAGCTTACTACTGAA-3′; shIGF2 BP3#1 targeted sequence, 5′-GCTGAGAAGTCGATTACTA-3′; shIGF2BP3#2 targeted sequence, 5′-TCGGAAACTTCAGATACGA-3′. si-P300#1 forward, 5′-CG UGUCUUCACUUAAGAGUdTdT-3′ and reverse, 5′-ACUCUUAAGUGAAGACA CGdTdT-3′; si-P300#2 forward, 5′-CAUACUUAGACGUUUGUUAdTdT-3′ and reverse, 5′-UAACAAACGUCUAAGUAUGdTdT-3′; si-CBP#1 forward, 5′-GUCAC CCUUGGAACAAGGUdTdT-3′ and reverse, 5′-UGGAACAAGGUUCCCACUGdT dT-3′; si-CBP#2 forward, 5′-CCUGACGUCAGAUGUACU dTdT-3′ and reverse, 5′-UCAUGAUAGACUGCAGUCCdTdT-3′; si-TMBIM6#1 forward, 5′-GUGGAAG GCCUUCUUUCUAdTdT-3′ and reverse, 5′-UAGAAAGAAGGCCUUCCACdTdT − 3′; si-TMBIM6#2 forward, 5′-UUCCGUGACGUAACUAGAGdTdT-3′ and reverse, 5′-CUCUAGUUACGUCACACGGAAdTdT-3′; and si-Ctrl forward, 5′-UUCUCCG AACGUGUCACGUTT-3′ and reverse, 5′-ACGUGACACGUUCGGAGAATT-3′.

### Cell proliferation assay

Cell proliferation was detected using CCK-8 assay (Beyotime Institute of Biotechnology, Shanghai, China) and Real-time Cellular Analysis (RTCA assay; ACEA Biosciences, Inc.; Agilent Technologies, Inc.), as previously described [23–25]. For CCK-8 assay, cells were transfected with the plasmids and seeded in 96-well plate at a density of 5, 000–10, 000 cells/well. Cells were cultured for 24, 48, and 72 hours, followed by incubation with 10 μl CCK-8 reagent, the absorbance values at 450 nm were determined by a Spectra Max 190 reader (Molecular Devices, LLC). For RTCA assay, 5, 000–10, 000 cells were seeded into the E-plate. The data were recorded using xCELLLigence software 2.0 (ACEA Biosciences, Inc.; Agilent Technologies, Inc.) and analyzed using GraphPad Prism 5.0 (GraphPad Software, Inc.).

### Transwell assay

The migratory and invasive abilities of NSCLC cells were analyzed using Transwell assay (Coring; pore, 8 μm; USA). In brief, cells were seeded in the upper chamber with serum-free medium, while the lower chamber contained complete medium to induce migration and invasion. Following the indicated time incubation, the migrated cells were fixed with 4% paraformaldehyde (PFA) and then stained with 1% crystal violet stain solution (Beyotime Institute of Biotechnology). For cell invasion, the upper chamber was firstly coated with Matrigel® Matrix Basement Membrane (cat. no. 356234; BD Biosciences, USA). The images were captured under an inverted fluorescence microscope (ZEISS Axio Vert.A1; Carl Zeiss AG). In addition, the stained cells were decolorized with 30% acetic acid, and the absorbance values were determined by a Spectra Max 190 reader (Molecular Devices, LLC) at 570 nm.

### Real-time cellular analysis (RTCA)

The RTCA xCELLLigence system (ACEA Biosciences, Inc.; Agilent Technologies, Inc.) was usually used to measure cell morphology, proliferation and migration in vitro in a non-invasive manner, as described previously [23–25]. In brief, the lower chamber was added into complete medium, while cells were seeded into serum-free culture medium to induce migration and invasion. The data were recorded using xCELLLigence software 2.0 (ACEA Biosciences, Inc.; Agilent Technologies, Inc.) and analyzed using GraphPad Prism 5.0 (GraphPad Software, Inc.).

### RNA extraction and RT-qPCR

Total RNA was extracted from tissues or cells using TRIzol® reagent (Invitrogen, Thermo Fisher Scientific, Inc.), according to the manufacturer’s instructions. 500 ng total RNA was reverse transcribed into cDNA using the PrimeScript RT reagent kit (Takara Biotechnology Co., Ltd.), as our previously described [24, 25]. RT-qPCR was performed using UltraSYBR Green PCR Master mix (cat. no. CW0957H; CWBIO, Beijing, China) on an ABI 7500 system (Applied Biosystems; Thermo Fisher Scientific, Inc.). The results were normalized to 18S ribosomal RNA (18sRNA), and the relative expression level was calculated using the 2^-ΔΔCq^ method [26]. Sequences of the primers are described in Additional file: Table S2.

### Western blot analysis

Total protein was extracted from tissues or cells using RIPA buffer (cat. no. P0013B; Beyotime Institute of Biotechnology) containing protease and phosphatase inhibitors (Beyotime Institute of Biotechnology). An equal amount of protein was loaded and separated with 8–12% SDS-PAGE. The protein was transferred onto a PVDF membrane (Invitrogen, Thermo Fisher Scientific, Inc.) and then incubated with primary antibodies at 4 °C overnight. The membrane was washed with phosphate buffer saline (PBS) containing 0.1% Tween-20 and incubated with corresponding second antibodies at room temperature for 1 hour. The images were obtained using the Tanon 5200 (Tanon).

### GST fusion protein pull-down assay

The GST-vector and GST-IGF2BP3 fusion protein plasmids were transfected into *Escherichia coli* BL-21 (DE3) cells. Fusion proteins were induced expression by isopropyl β-D-thiogalactoside (IPTG) and purified using the GST protein purification kit (Beyotime Institute of Biotechnology) according to the manufacturer’s protocol. The bound protein was identified using Coomassie staining. GST fusion protein solutions were incubated with beads at 4 °C for 3 h. Whole 293 T cell lysates transfected with STRIP2-flag protein were incubated with beads containing GST fusion proteins at 4 °C overnight. The beads were washed and the protein was detected using western blotting assay.

### Immunofluorescence

Cells seeded on coverslips were fixed in 4% PFA for 30 min at room temperature followed by permeabilization with 0.1% of Triton-X-100 (Beyotime Institute of Biotechnology) in PBS for 20 min at room temperature. After three time washing, fixed cells were then blocked with 5% Bovine Serum Albumin (BSA) in PBS for 1 hour at room temperature and incubated with a polyclonal rabbit anti-STRIP2 antibody (1:200, cat. no. PA5–54047, Invitrogen, Thermo Fisher Scientific, Inc.) at 4 °C overnight in darkness. Cells were washed with PBS and then incubated with goat-anti rabbit secondary antibody conjugated to Alexa Fluor 488 (Thermo Fisher Scientific, Inc.) for 1 hour at room temperature in darkness. Cells were stained using antifade solution containing DAPI and mounted on an inverted fluorescence microscope (ZEISS Axio Vert.A1; Carl Zeiss AG).

### Immunohistochemistry staining

IHC staining was performed as described previously [25]. In brief, slides were incubated with anti-STRIP2 antibody (dilution 1:200), anti-IGF2BP3 antibody (dilution 1:200) and anti-TMBIM6 antibody (dilution 1:200) at 4 °C overnight and then cultured with SP Rabbit & Mouse HRP-conjugated secondary antibody (cat no. CW2096S; CWBio; https://www.cwbio.com/) for 1 h at room temperature. And the slides were incubated with DAB kit (cat. no. CW2096S; CWBio) following counterstained with hematoxylin staining solution (cat. no. C0107; Beyotime Institute of Biotechnology). The images were digitally scanned at a magnification of × 400 using a KF-PRO-005 platform (Ningbo Jiangfeng Bio-information Technology Co., Ltd.) into whole slide digital images. The scoring of the slides was conducted using HALO Multiplex IHC analysis software version v3.1.1076.308 (Indica Labs). The intensity was scored as 1 (absent or weak), 2 (moderate) or 3 (high). The percentage of positive cells was assigned as 1 (< 25%), 2 (25–50%) and 3 (> 50%). The score of each slice was multiplied to acquire a final score of 1–9.

### Methylated RNA immunoprecipitation-PCR (MeRIP-qPCR)

The MeRIP-qPCR assay was conducted using the ribo*MeRIP*™ m6a Transcriptome Profiling kit (Guangzhou RiboBio Co., Ltd.; Guangzhou), according to the manufacturer’s instructions. In brief, total RNA was extracted with TRIzol® reagent (Invitrogen, Thermo Fisher Scientific, Inc.) and RNA was fragmented using the RNA fragmentation buffer supplied with the kit. The specific anti-m6A antibody was vortically pre-bound to Protein A/G magnetic beads at room temperature for 30 min. Then the fragmented RNA was incubated with m6A-antibody-bound Protein A/G magnetic beads at 4 °C for 2 h and washed twice using wash buffer. The m6A-antibody-bound RNA was purified using GeneJET RNA Purification kit (Invitrogen, Thermo Fisher Scientific, Inc.). Real-time PCR was performed following m6A-IP to quantify the changes to m6A methylation of TMBIM6. The primer sequences used in the experiment were as follow: *TMBIM6* 5′-TCATATAACCCCGTCAACGC-3′ (sence) and 5′-CAAATCCAGCAAGAAGTC CC-3′ (antisence).

### Chromatin Immunoprecipitation (CHIP) assay

CHIP assay was carried out using the SimpleChIP® Enzymatic Chromatin IP kit (Magnetic Beads) (cat. no. 9003; Cell Signaling Technology) according to the manufacturer’s protocol. Briefly, about 5 × 10^7^ NSCLC cells were cross-linked with 1% formaldehyde for 10 min at room temperature. The cross-linking was quenched by addition of 125 mM glycine for 5 min at room temperature and followed by sonication. The fragmented chromatin was analyzed on agarose gels. After purifying, the chromatin was incubated with specific CBP antibody (cat. no. 7425; Cell Signaling Technology) or P300 antibody (cat. no. 54062; Cell Signaling Technology) to immunoprecipitation chromatin overnight at 4 °C with rotation and followed by incubation with protein G magnetic beads for 2 h at 4 °C. The eluted DNA was purified according to the kit and then used as template for RT-qPCR with specific primers: *STRIP2* (promoter), 5′-CCCAGTCATGGGCTGCA ATAA-3′ (sense) and 5′-CTGAGCACAGAAGTCACCAGTT-3′ (antisense). The control primers for human RPL30 were provided with the kit. The process of RT-qPCR was carried out according to described previously [27, 28].

### RNA and protein stability assays

To explore the stability of TMBIM6 mRNA and protein under the influence of downregulation or upregulation of STRIP2/IGF2BP3, cells were treated with actinomycin D (5 μg/ml; cat. no. HY-17559; MedChemExpress) for 0, 6, 12, 24 h. The procedures of total RNA isolation and RT-qPCR were performed as described previously [29].

### RNA sequencing

Total RNA was harvested and isolated from the stable STRIP2 knockdown and scramble control cells. The total RNA was then performed to ribosomal RNA depleted RNA sequencing (RNA-Seq) protocols as previously described [30, 31]. The library was sequenced on an Illumina Novaseq 6000. Differentially expressed genes were defined as fold change > 2 or fold change < 0.5 and *p* < 0.05, and then Gene Ontology (GO) and Kyoto Encyclopedia of Genes and Genomes (KEGG) pathway enrichment analyses were performed. In addition, the venn diagram was conducted using STRIP2 knockdown sequence data and two published IGF2BP3 RIP-sequence data and GEO database (GSE90684). All protocols and analyses were provided by LC Biotech Corporation (Hangzhou, China).

### Luciferase reporter assay

Luciferase reporter assay was performed as previous study [29]. In brief, cells were transfected with a luciferase reporter containing TMBIM6 WT or Mut and Renilla plasmids. Luciferase activities were detected using a Dual-Luciferase Reporter Gene Assay Kit (Beyotime Institute of Biotechnology).

### Cell apoptosis assay

Cell apoptosis assay was carried out using the Annexin-V/propidium iodide (PI) detection kit (cat. no. PF00005; Proteintech Group, Inc.,), according to the manufacturer’s protocol. In briefly, cells were transfected with scrambled siRNA and two specfic siRNAs of TMBIM6 for 24 hours. After transfection, the cells were harvested and washed with phosphate buffer saline (PBS) twice and then resuspended in a binding buffer containing Annexin-V and PI for 15 min on ice in darkness. Cells were measured using the BD FACSCanto II system (BD Biosciences).

### Statistical analysis

All data were represented as mean ± SEM of three independent experiments and analyzed using SPSS software version 21.0 (IBM Corp.) with an unpaired Student’s t-test or one-way ANOVA followed by a Tukey’s post hoc test. The associations between STRIP2 expression and patient clinicopathological features of patients were analyzed using the χ^2^ test and Fisher’s exact test. The correlations of STRIP2, IGF2BP3 and TMBIM6 expression were analyzed using Pearson rank correlation analysis. Kaplan-Meier survival analysis was performed using the log-rank test. *P* value< 0.05 was considered statistically significant.

## Results

### STRIP2 is highly expressed in NSCLC

**Fig. 1:**
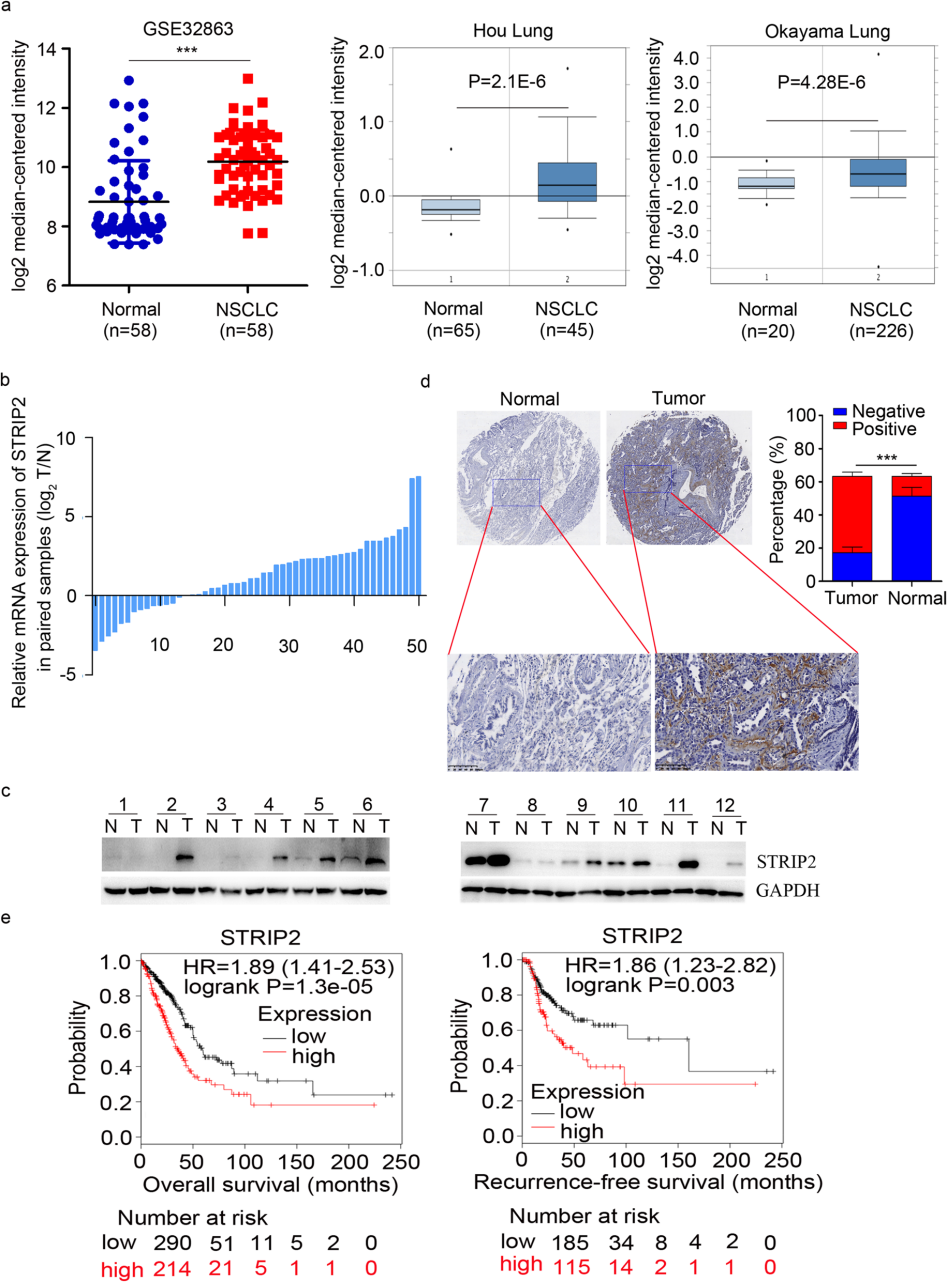
STRIP2 is elevated in NSCLC and predicts poor survival. **a** STRIP2 mRNA expression was investigated in GSE32863 database and two published datasets form Hou Lung and Okayama Lung. **b** RT-qPCR analysis of STRIP2 mRNA in 51 paired NSCLC and normal tissues. **c** Western blot analysis of STRIP2 expression in 12 paired normal (N) and NSCLC (T) tissues. GAPDH is used as internal control. **d** Immunohistochemistry analysis of STRIP2 in a NSCLC tissue microarray containing 189 paired normal and NSCLC tissues. Scale bar, 100 μm. **e** Kaplan-Meier analysis of overall survival data from TCGA NSCLC datasets containing 601 patients and recurrence-free survival data from TCGA NSCLC datasets containing 366 patients. All data were presented as the mean ± SEM

To explore the expression pattern of STRIP2 in NSCLC, we firstly analyzed the GEO database (GSE32863) and found that the mRNA levels of STRIP2 were upregulated in NSCLC (Fig. 1a). Further, in silico analysis of two independent datasets from Oncomine (Hou Lung, *n* = 110 and Okayama Lung, *n* = 246) demonstrated that STRIP2 mRNA levels were significantly higher in NSCLC than their corresponding normal counterparts (Fig. 1a). To confirm the results of datasets, we quantified the mRNA level of STRIP2 in 51 paired NSCLC tissues and their match adjacent normal lung tissues by reverse transcription-quantitative PCR (RT-qPCR). Upregulation of STRIP2 was observed in 37 pairs, accounting for 73% of total samples examined (Fig. 1b). Consistently with the mRNA expression pattern, an increased protein expression level of STRIP2 was observed in NSCLC tissues compared with their matched adjacent normal lung tissues in 10 out of 12 paired samples, as determined using western blot analysis (Fig. 1c). Elevated expression of STRIP2 protein in NSCLC was further confirmed by immunohistochemistry analysis (Fig. 1d). Collectively, these data suggested that STRIP2 expression was increased in NSCLC.

### Clinical significance of STRIP2 expression in NSCLC

To evaluate the clinical significance of STRIP2 in NSCLC, a tissue microarray (TMA) containing 189 NSCLC samples and their match noncancerous lung samples was performed to detect the expression levels of STRIP2 by immunohistochemistry staining. The values were scored in a standard manner as described previously [25]. In total, 138 NSCLC samples were classified into the high STRIP2 expression group, while 51 samples were classified into the low STRIP2 expression group (Fig. 1d and Table S1). Further correlation analysis demonstrated that higher STRIP2 levels were correlated with clinicopathological features of patients with NSCLC including poor tumor differentiation (*P* = 0.041), advanced TNM stage (*P* = 0.038), positive of lymph node metastasis (*P* = 0.026) and positive of cancer thrombus (*P* = 0.045) (Table S1). However, there was no association with sex, age, smoking history, tumor size and histological type. Moreover, NSCLC patients with high STRIP2 expression manifested a shorter overall survival (*n* = 242, HR = 1.89, *P* = 1.3e-5) and recurrence-free survival (*n* = 133, HR = 1.86, *P* = 0.003) than patients with low STRIP2 expression (Fig. 1e).

### P300/CBP-mediated H3K27ac promotes STRIP2 transcription in NSCLC

**Fig. 2:**
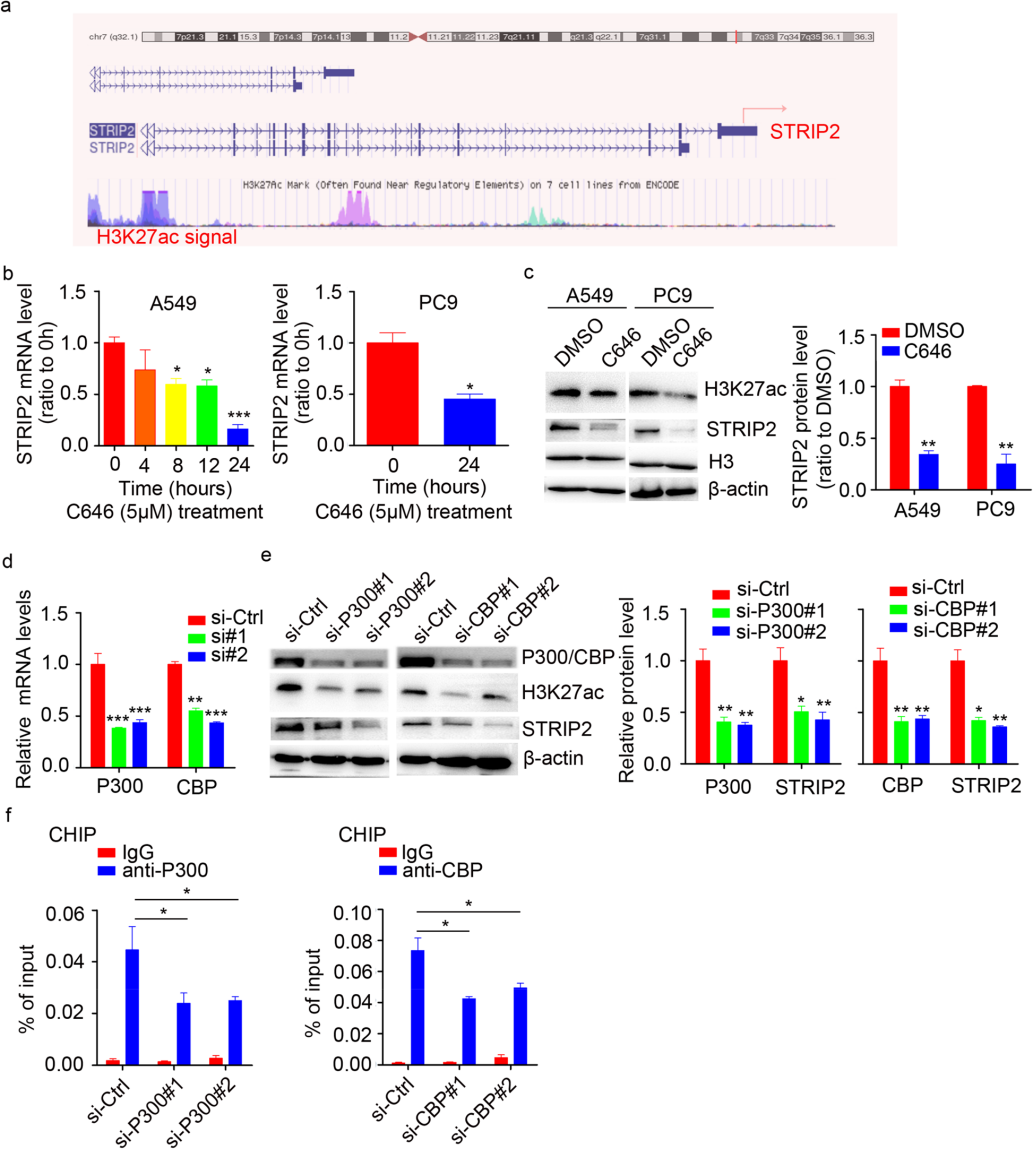
P300/CBP-mediated H3K27ac activates STRIP2 transcription in NSCLC. **a** Data from the UCSC genome bioinformatics site (http://genome.ucsc.edu/) showed high enrichment of H3K27ac in the promoter of STRIP2. **b** RT-qPCR analysis of STRIP2 mRNA level in C646 (5 μM)-treated A549 and PC9 cells at the indicated time point. **c** STRIP2 protein levels were measured by western blotting after C646 (5 μM)-treated A549 and PC9 cells for 24 hours. **d** The efficiency of P300 and CBP knockdown was performed using RT-qPCR. **e** The P300, H3K27ac and STRIP2 levels were detected using western blotting after P300 or CBP knockdown. **f** CHIP assay were used to measure the level of P300 or CBP binding at the promoter of STRIP2 in P300 or CBP deficiency or control A549 cells. Data represent mean ± SEM from three independent experiments. *, *p* < 0.05; **, *p* < 0.01 were determined by one-way ANOVA with Tukey’s post hoc analysis. H3K27ac, H3K27 acetylation; CHIP, chromatin immunoprecipitation

To explore the molecular mechanism of high STRIP2 expression in NSCLC, we first analyzed the modification in the promoter of STRIP2 by the USCS genome bioinformatics site (http://genome.ucsc.edu/). As shown in Fig. 2a, plentiful H3K27 acetylation (H3K27ac) signals were observed in the promoter region of STRIP2, suggesting that STRIP2 might be regulated by chromatin acetylation. Increasing evidence has demonstrated that H3K27ac is catalyzed by the P300/CBP complex. The expression levels of P300 and CBP were significantly increased in NSCLC by analysis of GEO dataset (GSE32863) (Additional files Fig. S1). Furthermore, A549 and PC9 cells were treated with C646, a histone acetyltransferase inhibitor targeting P300/CBP, and the results showed that the expression of STRIP2 was decreased in a time-dependent and dose-dependent manner, which did not show no obvious cytotoxicity in A549 and PC9 cells (Fig. 2b; Additional files Fig. S2). Consistently with the mRNA expression pattern, the protein level of STRIP2 was significantly downregulated after C646 treatment (Fig. 2c). Next, we knocked down the endogenous P300 by two specific siRNAs, and suggested that knockdown of P300 significantly reduced the mRNA and protein levels of STRIP2 (Fig. 2d and e). This result was also observed in the knockdown of CBP (Fig. 2d and e). Moreover, the chromatin immunoprecipitation (CHIP) assay results demonstrated that downregulation of P300 and CBP could significantly decrease the enrichment of H3K27ac signals in the promoter of STRIP2 (Fig. 2f). Taken together, these data indicated that P300/CBP-mediated H3K27ac activation in the promoter of STRIP2 may partly explain the upregulation of STRIP2 in NSCLC.

### STRIP2 regulates cell tumorigenic phenotype in vitro

**Fig. 3:**
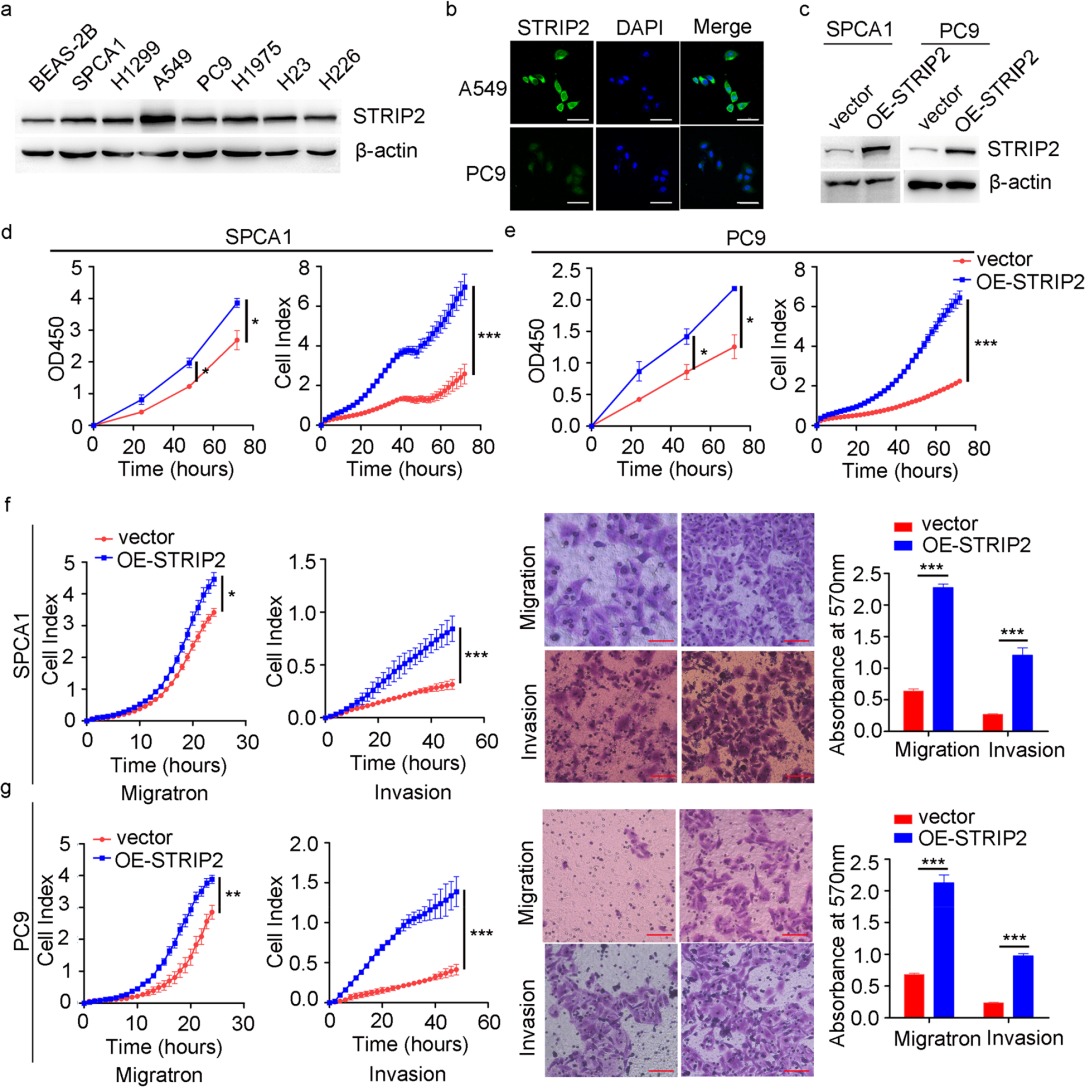
Overexpression of STRIP2 promotes cell proliferation, migration and invasion in vitro. **a** The expression of STRIP2 in seven NSCLC cells were detected using western blotting. **b** Immunofluorescence assay analysis of STRIP2 level in A549 and PC9 cells. **c** The efficiency of STRIP2 overexpression was verified at the protein levels in SPCA1 and PC9 cells by western blotting. **d** Cell viability was analyzed using RTCA and CCK8 assays in SPCA1 cells. **e** Cell viability was analyzed using RTCA and CCK8 assays in PC9 cells. **f** Cell migratory and invasive abilities were detected using RTCA and Transwell assays in SPCA1 cells. **g** Cell migratory and invasive abilities were detected using RTCA and Transwell assays in PC9 cells. Data represent mean ± SEM from three independent experiments. *, *p* < 0.05; **, *p* < 0.01; ***, *p* < 0.001 were determined by one-way ANOVA with Tukey’s post hoc analysis. OE, overexpression

The expression of STRIP2 in NSCLC cells was firstly measured by western blotting, and the results indicated that STRIP2 was highly expressed in NSCLC cells compared to human normal bronchial epithelial cell line (BEAS-2B) (Fig. 3a). In addition, immunofluorescent assay demonstrated that STRIP2 was mainly expressed in cytoplasm (Fig. 3b). Considering the associations between STRIP2 expression and NSCLC progression in clinic, we overexpressed it in both SPCA1 and PC9 cells (Fig. 3c), which showed relatively low expression of STRIP2 (Fig. 3a). Furthermore, the CCK-8 and RTCA assays showed that overexpression of STRIP2 promoted SPCA1 cell proliferation in a time-dependent manner (Fig. 3d), and this result was also observed in PC9 cell (Fig. 3e). Moreover, the Transwell and RTCA assays indicated that STRIP2 overexpression significantly increased cell migratory and invasive capabilities in both SPCA1 and PC9 cells (Fig. 3f and g).

**Fig. 4:**
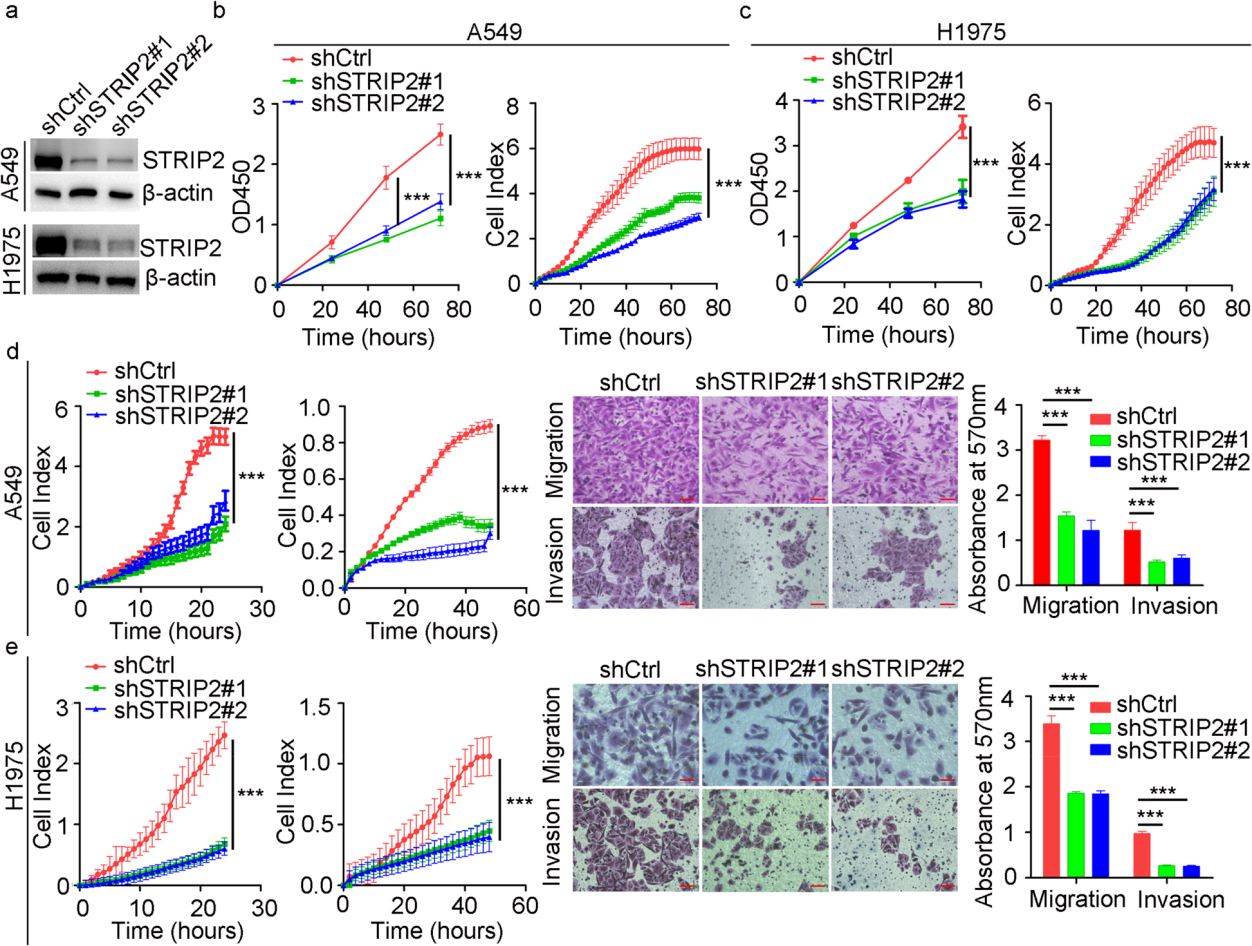
STRIP2 knockdown inhibits cell proliferation, migration and invasion in vitro. **a** The efficiency of STRIP2 knockdown in A549 and PC9 cells was detected using western blotting. **b** Cell viability was analyzed using RTCA and CCK8 assays in A549 cells. **c** Cell viability was analyzed using RTCA and CCK8 assays in H1975 cells. **d** Cell migratory and invasive abilities were detected using RTCA and Transwell assays in A549 cells. **e** Cell migratory and invasive abilities were detected using RTCA and Transwell assays in H1975 cells. Data represent mean ± SEM from three independent experiments. *, *p* < 0.05; **, *p* < 0.01; ***, *p* < 0.001 were determined by one-way ANOVA with Tukey’s post hoc analysis

In addition, we established stable STRIP2 knockdown NSCLC cells (A549 and H1975, two NSCLC cells with relative high expression level of STRIP2) (Fig. 4a). As expected, downregulation of STRIP2 was obviously reduced cell proliferative, migratory and invasive capabilities of NSCLC cells compared to the control cells using CCK-8, Transwell and RTCA assays (Fig. 4b-e). These results revealed that STRIP2 regulates NSCLC cell proliferation, migration and invasion in vitro.

### STRIP2 reduces tumor growth and metastasis in vivo

**Fig. 5:**
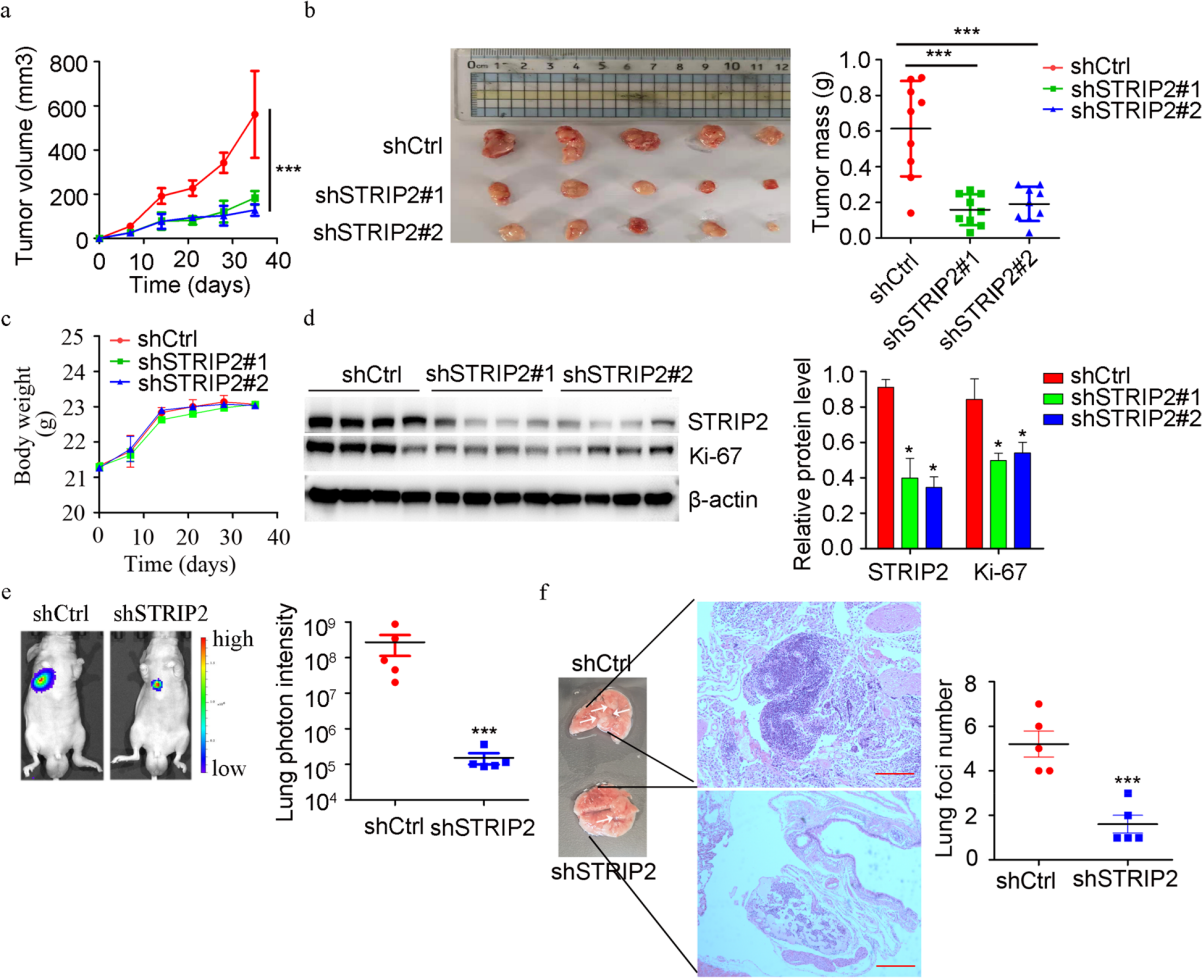
Knockdown of STRIP2 suppresses tumor growth and metastasis in vivo. **a** Effects of A549 knockdown cells on tumor growth in subcutaneously implanted BALB/c mice (*n* = 9). **b** Endpoint tumor size and mass in BALB/c mice (*n* = 9). **c** Effects of A549 knockdown cells on body weight in BALB/c mice. d STRIP2 and Ki-67 expressions were detected using western blotting. **e** Representative images showing luciferase expression from lung metastasis of both A549 control and shSTRIP2 group. **f** HE staining of lungs from both control and shSTRIP2 groups resected from tail vein injection metastasis mouse model. *, *p* < 0.05; **, *p* < 0.01; ***, *p* < 0.001 were determined by one-way ANOVA with Tukey’s post hoc analysis

To assess the effect of STRIP2 in vivo, subcutaneous transplantation assay and tail vein metastasis assay were performed. In subcutaneous transplantation assay, A549-luciferase cells knockdown STRIP2 and the corresponding control cells were subcutaneously injected (*n* = 9 mice in each group), respectively. After 5 weeks, downregulation of STRIP2 significantly suppressed tumor growth and mass (Fig. 5a-b) compared with those in the control group. But, STRIP2 knockdown didn’t have a significant effect on body weight of mice (Fig. 5c). In addition, western blotting analysis indicated that STRIP2 and Ki-67 expressions were reduced in STRIP2 knockdown group (Fig. 5d). In the tail vein assay, STRIP2 knockdown and control A549 cells were injected into the tail veins of nude mice (*n* = 5 for each group) to determine their ability of lung colonization, and the results suggested that the lung luciferase signals of STRIP2 knockdown group were significantly lower compared with those in the corresponding control group (Fig. 5e). Moreover, HE staining showed that number of metastatic nodules in the lungs was decreased in the STRIP2 knockdown group (Fig. 5f). Taken together, these data suggest downregulation of STRIP2 reduces tumor growth and metastasis in vivo.

### STRIP2 and IGF2BP3 cooperate to play oncogenic roles in NSCLC

**Fig. 6:**
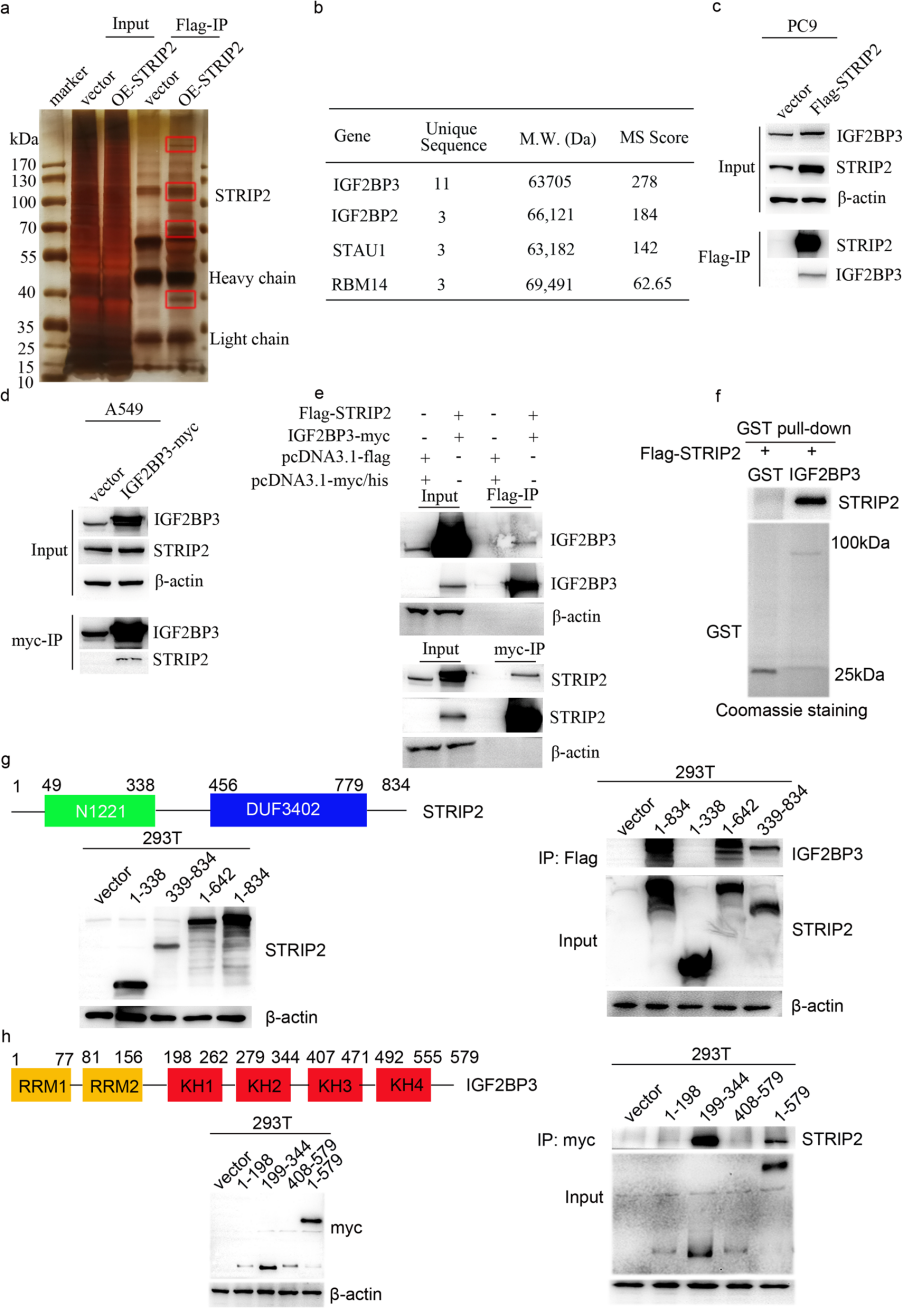
STRIP2 interacts with IGF2BP3. **a** Silver stained gel shows differential bands between control and STRIP2 samples. Red box indicates differential bands. **b** Tandem mass spectrum analysis of target band. **c** Interaction between STRIP2 and endogenous IGF2BP3 was demonstrated using immunoprecipitation. PC9 cells were transfected with Flag-STRIP2. **d** Interaction between endogenous STRIP2 and IGF2BP3 was demonstrated using immunoprecipitation. A549 cells were transfected with IGF2BP3-myc. **e** Interaction between STRIP2 and IGF2BP3 was demonstrated using immunoprecipitation. 293 T cells were concurrently transfected with Flag-STRIP2 and IGF2BP3-myc. **f** In vitro interaction between STRIP2 and IGF2BP3 was conducted by GST-pulldown assay. GST protein was identified using Coomassie staining. **g** The domain of STRIP2 (339–834) was interacted with IGF2BP3 using immunoprecipitation. **h** The domain of IGF2BP3 (199–407) was interacted with STRIP2 using immunoprecipitation

To further elucidate the molecular mechanisms of STRIP2 in NSCLC, we performed immunoprecipitation assay coupled with mass spectrometry to screen *STRIP2*-interacting proteins (Fig. 6a), and the results indicated that IGF2BP3 is a putative *STRIP2*-binding protein (Fig. 6b and Additional files Table S3). In addition, immunoprecipitation assay confirmed that STRIP2 was strongly interacted with endogenous IGF2BP3 (Fig. 6c). We also found that IGF2BP3 could interact with endogenous STRIP2 (Fig. 6d). Furthermore, we overexpressed 3xFlag-STRIP2 and IGF2BP3-myc in 293 T cells, and the immunoprecipitation assay indicated that STRIP2 interacted with IGF2BP3 (Fig. 6e). To examine whether this interaction was direct or indirect, we performed GST pull-down assay. The results revealed that IGF2BP3 could directly bind to STRIP2 (Fig. 6f). We further explore which domain of STRIP2 required for this interaction. As shown in Fig. 6g, truncated mapping indicated that the 339–834 nt region within *STRIP2* interacts with IGF2BP3. IGF2BP3 mainly contained two RNA-recognition-motif (RRM) domains and four homology (KH) domains (Fig. 6h). Immunoprecipitation assay for myc-tagged full length and truncated IGF2BP3 demonstrated that the KH1 and KH2 domains were essential for this interaction (Fig. 6h). Overall, these data indicate that STRIP2 directly interacts with IGF2BP3.

**Fig. 7:**
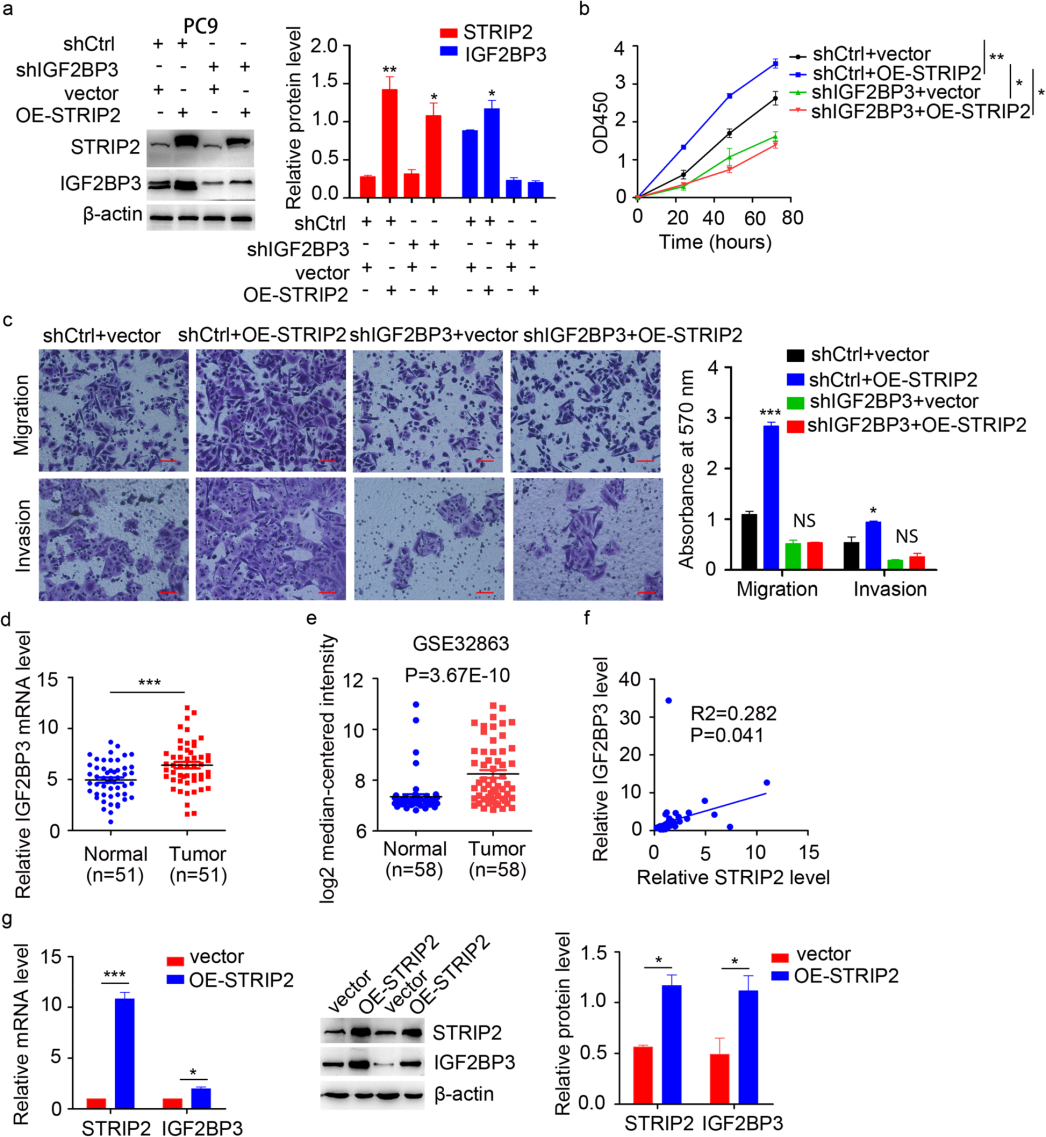
STRIP2 promotes tumor progression dependent on IGF2BP3 in NSCLC. **a** IGF2BP3 level was detected using western blotting and statistically analyzed in IGF2BP3 knockdown PC9 cells with STRIP2 overexpression. **b** Cell viability was measured using CCK-8 assay. **c** Cell migratory and invasive abilities were detected using Transwell assay. **d** RT-qPCR analysis of IGF2BP3 mRNA level in 51 paired NSCLC and normal tissues. **e** IGF2BP3 mRNA expression was investigated in GSE32863 database and compared between normal (*n* = 58) and NSCLC (n = 58) tissues. **f** Correlation analysis between STRIP2 expression and IGF2BP3 level. g IGF2BP3 mRNA and protein levels were detected using quantitative RT-PCR and western blotting in STRIP2 overexpression. Data represent mean ± SEM from three independent experiments. *, *p* < 0.05; **, *p* < 0.01; ***, *p* < 0.001 were determined by one-way ANOVA with Tukey’s post hoc analysis. OE, overexpression

To evaluate whether IGF2BP3 was required for the effect of STRIP2 on NSCLC progression, we knocked down IGF2BP3 expression in PC9 cells, and then overexpressed STRIP2 in these cells (Fig. 7a). Strikingly, IGF2BP3 knockdown abrogated the promoting effects of STRIP2 overexpression on cell proliferation, migration and invasion (Fig. 7b-c). To further explore the correlation between STRIP2 and IGF2BP3, we firstly detected the expression of IGF2BP3 in 51 pairs of NSCLC tissues, and the data shown that IGF2BP3 was highly expressed in NSCLC (Fig. 7d). This result was confirmed by the GEO database (GSE32863) (Fig. 7e). Correlation analysis demonstrated that STRIP2 expression was positively correlated with IGF2BP3 level (Fig. 7f). In addition, quantitative RT-PCR and western blotting assay showed that overexpression of STRIP2 increased IGF2BP3 protein level through increasing the transcription of IGF2BP3 (Fig. 7g). To certify whether IGF2BP3 regulated STRIP2 expression, cells were transfected with IGF2BP3 plasmids and the results indicated that overexpression or knockdown of IGF2BP3 didn’t affect the STRIP2 mRNA and protein levels (Additional files Fig. S3a-c). Taken together, STRIP2 promotes NSCLC progression dependent on IGF2BP3.

### STRIP2 cooperates with IGF2BP3 to stabilize TMBIM6 via m6A

**Fig. 8:**
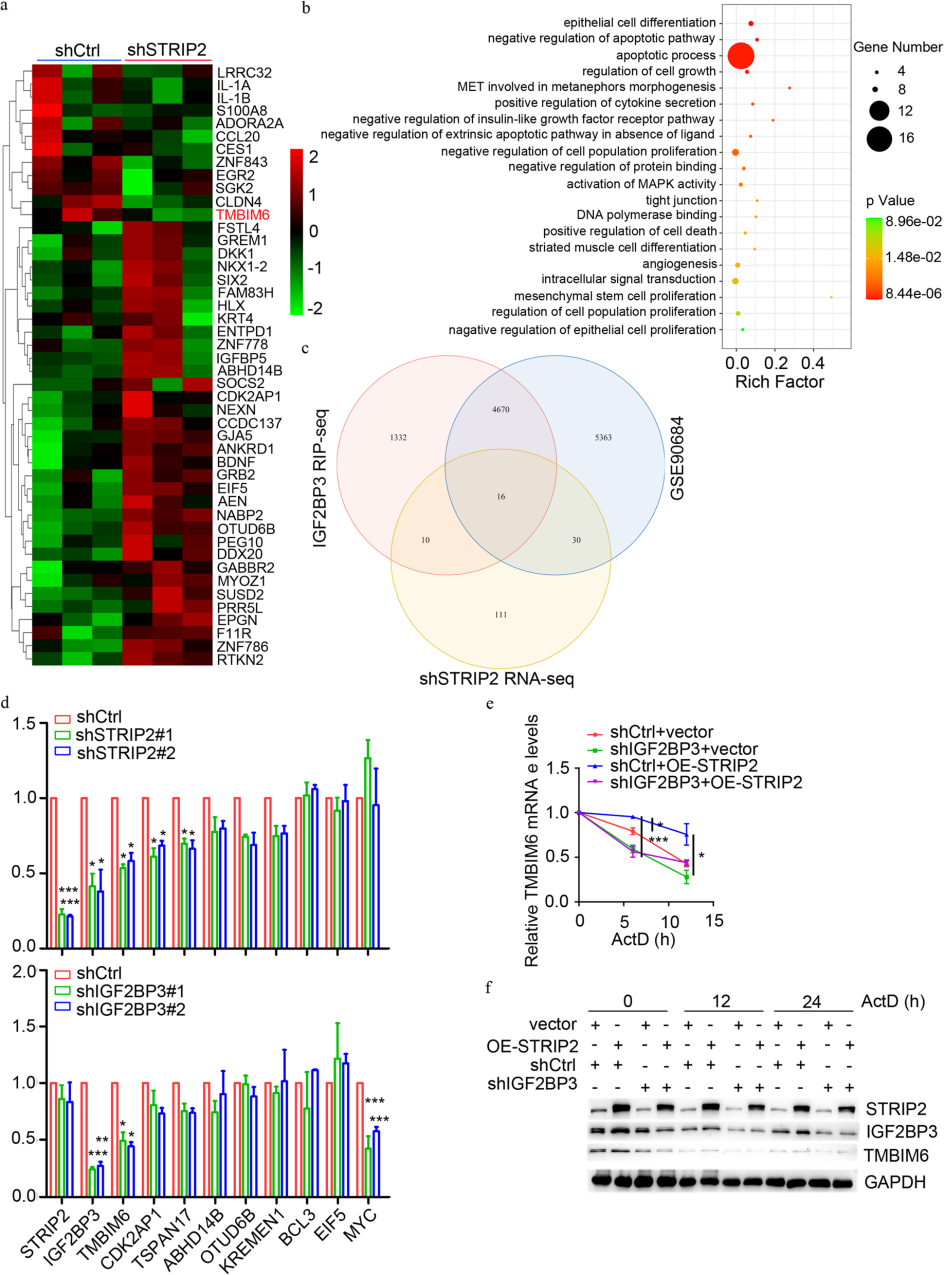
STRIP2 cooperates with IGF2BP3 to stabilize *TMBIM6* mRNA. **a** Heat map shows differential gene expression between shCtrl and shSTRIP2 samples. **b** The top 20 enriched GO terms for differentially expressed genes (DEGs). **c** Venn diagram showing the 16 overlapping genes of STRIP2 knockdown RNA-sequence data and IGF2BP3 RIP-sequence and GEO database (GSE90684). **d** RT-qPCR analysis of 8 overlapping genes and *MYC* transcript levels in the STRIP2 knockdown and IGF2BP3 knockdown A549 cells. **e**-**f** The half-life of *TMBIM6* after treatment with 5 μg/ml actinomycin D for the indicated times in the IGF2BP3 knockdown PC9 cells with STRIP2 overexpression. Data represent Mean ± SEM from three independent experiments. *, *p* < 0.05; ***, *p* < 0.001 were determined by one-way ANOVA with Tukey’s post hoc analysis. OE, overexpression

Considering IGF2BP3 as an mRNA binding protein, which stabilizes a large repertoire of target mRNA transcripts, we hypothesized that STRIP2-IGF2BP3 axis regulates NSCLC progression by modulating the targeted gene mRNA stability. To test this hypothesis, we performed an integrated analysis on the RNA-sequence data of STRIP2 knockdown and two published IGF2BP3 RIP-sequence data and GEO database (GSE90684) to discover the target genes potentially regulated by STRIP2 and IGF2BP3. Gene expression profiling of STRIP2 knockdown demonstrated that upregulation of 87 genes and downregulation of 168 genes were obtained (Additional files Fig. S4 and Table S4). These genes were mainly enriched in cell apoptotic process, cell proliferation and tight junction (Fig. 8a and b). Among 168 downregulated genes after STRIP2 knockdown, 16 genes had IGF2BP3’s enrichment on their transcripts through two published IGF2BP3 RIP-sequence data and GEO database (GSE90684) (Fig. 8c and Table S5). To certify the possible key targets of the STRIP2/IGF2BP3 axis, 8 genes (the most changed) were elected and proved using RT-qPCR assay. The results showed that the mRNA level of TMBIM6 was significantly decreased in knockdown of both STRIP2 and IGF2BP3, suggesting that TMBIM6 is co-target gene of STRIP2 and IGF2BP3 (Fig. 8d). As previous reports, [21, 32, 33] knockdown of IGF2BP3 was decreased the mRNA level of MYC, while STRIP2 knockdown was not influenced MYC expression (Fig. 8d). To further investigate whether STRIP2-IGF2BP3 axis regulates the stabilization of TMBIM6 mRNA, RNA stability assay was performed and the result indicated that IGF2BP3 knockdown shortened the half-life and mRNA level of TMBIM6 increased by the overexpression of STRIP2 (Fig. 8e). Consistent with the mRNA of TMBIM6, knockdown of IGF2BP3 reduced the TMBIM6 protein level and shortened the half-life of TMBIM6 increased by the overexpression of STRIP2 (Fig. 8f). Collectively, these data suggested that STRIP2 cooperates with IGF2BP3 to regulate TMBIM6 by enhancing its stability.

**Fig. 9:**
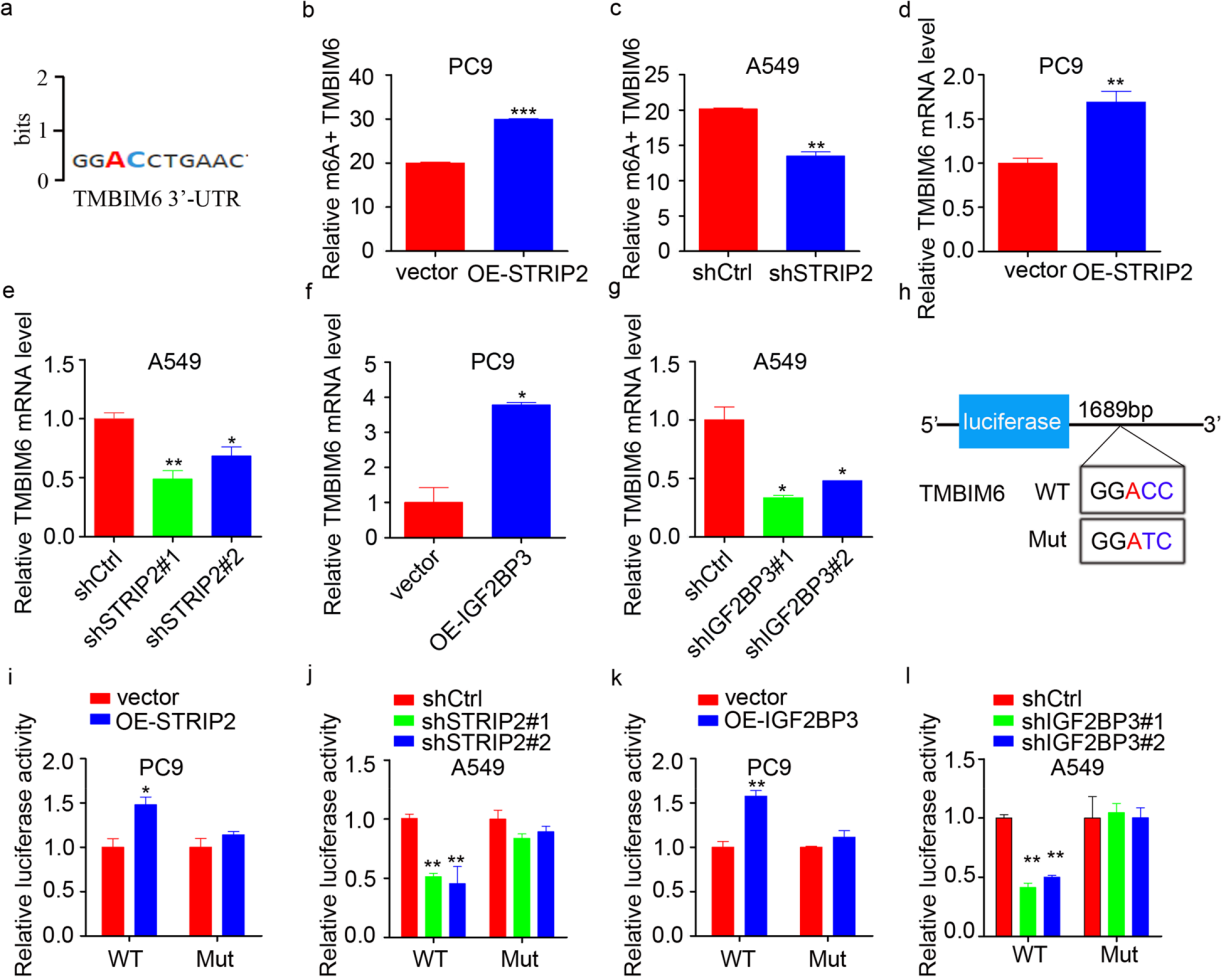
STRIP2 cooperates with IGF2BP3 to stabilize *TMBIM6* mRNA via m6A. **a** Data from the RMVar bioinformatics site (https://rmvar.renlab.org/) showed exists m6A site in the 3′-UTR of *TMBIM6*. **b**-**c** RIP-PCR analysis detecting the enrichment of TMBIM6 m6A modifications. **d**-**g** RT-PCR analysis the mRNA of TMBIM6. h Construction of TMBIM6-WT and Mut luciferase reporter plasmids. **i**-**l** Dual-luciferase activity in cells co-transfected with TMBIM6-WT or TMBIM6-Mut together with STRIP2 or IGF2BP3 overexpression and knockdown was measured. Data represent Mean ± SEM from three independent experiments. *, *p* < 0.05; **, *p* < 0.01; ***, *p* < 0.001 were determined by one-way ANOVA with Tukey’s post hoc analysis. OE, overexpression

As previous studies, [34–36] IGF2BP3 has been recognized as an m6A reader that regulates m6A-modified genes, which led us to hypothesize that STRIP2 regulates IGF2BP3-dependent gene in an m6A-denpendent manner. To test this hypothesis, we first identified the potential m6A-modified site of TMBIM6 based on RMVar database (https://rmvar.renlab.org/) and the results showed that the 3′-UTR of *TMBIM6* has an m6A site (Fig. 9a). In addition, knockdown of STRIP2 inhibited the content of m6A positive TMBIM6 level, while STRIP2 overexpression obtained the opposite effect (Fig. 9b and c). Consistent with the previous report, [29] IGF2BP3 downregulation or upregulation affected the content of m6A positive TMBIM6 level (Additional files Fig. S5). To further confirm whether STRIP2 and IGF2BP3 regulated TMBIM6 expression, quantitative RT-PCR assay indicated that overexpression of STRIP2 or IGF2BP3 increased the mRNA expression level of TMBIM6, while knockdown of STRIP2 or IGF2BP3 obtained the opposite effect (Fig. 9d-g). Moreover, according to the m6A site of TMBIM6 located at base 1689 of the 3′-UTR based on RMVar database (https://rmvar.renlab.org/), we mutated ACC in the base sequence to ATC and then carried out a luciferase assay (Fig. 9h). Dual-luciferase reporter assay was performed and the results demonstrated that the luciferase activity of OE-STRIP2 group was significantly higher compared to the vector group, while the activity of the TMBIM6-Mut group showed not obviously change (Fig. 9i). Similarly, in A549 cells, the activity of shSTRIP2 group was significantly lower compared to the shCtrl group, while the activity of the TMBIM6-Mut group did not significantly change (Fig. 9j). These similar results were mutually obtained in OE-IGF2BP3 and shIGF2BP3 groups (Fig. 9k-l). Taken together, these data demonstrate that STRIP2 cooperates with IGF2BP3 to regulate TMBIM6 in an m6A-dependent manner.

### STRIP2 and IGF2BP3 coordinate NSCLC progression partially through TMBIM6

**Fig. 10:**
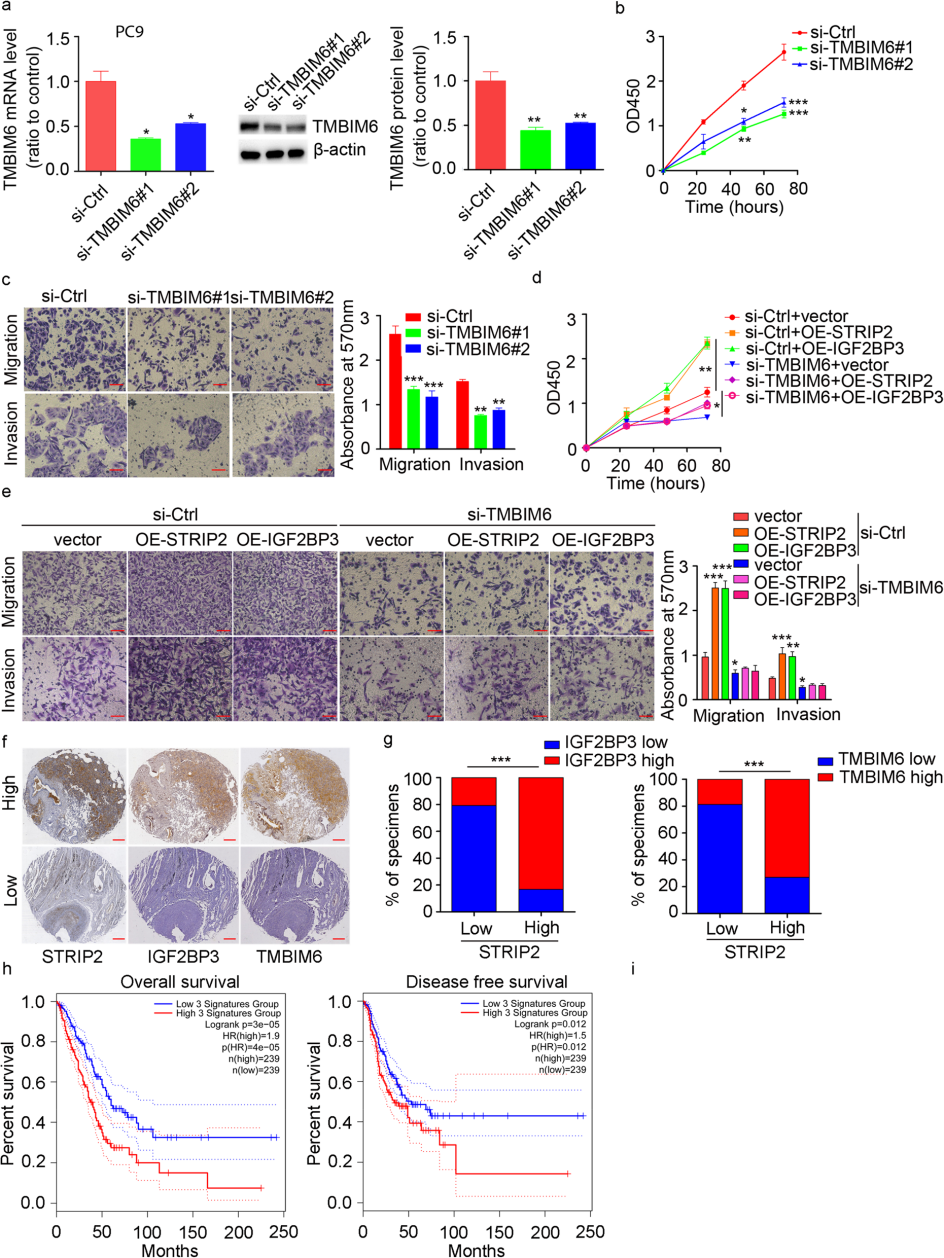
STRIP2 cooperates with IGF2BP3 to promote NSCLC progression dependent on TMBIM6. **a** The efficiency of si-TMBIM6 was analyzed using western blotting and quantitative RT-PCR in PC9 cells. **b** Cell proliferation was detected using CCK-8 after TMBIM6 knockdown in PC9 cells. **c** Cell migratory and invasive abilities were measured by Transwell assay. **d** Cell viability was performed using CCK-8 and statistically analyzed in IGF2BP3 or STRIP2 overexpression PC9 cells with TMBIM6 knockdown. **e** Cell migratory and invasive abilities were measured using Transwell assay. **f** Immunohistochemistry staining of STRIP2, IGF2BP3 and TMBIM6 in a NSCLC tissue microarray containing 48 paired normal and NSCLC tissues. Scale bar, 100 μm. g The percent of samples showing low or high STRIP2 expression relative to the levels of IGF2BP3 and TMBIM6 are shown. **h** Kaplan-Meier analysis of overall survival and disease-free survival data from TCGA NSCLC datasets containing 601 patients. Data represent mean ± SEM from three independent experiments. *, *p* < 0.05; **, *p* < 0.01; ***, *p* < 0.001 were determined by one-way ANOVA with Tukey’s post hoc analysis. OE, overexpression

TMBIM6, known as Bax inhibitor-1, was overexpressed and played oncogene roles in multiple cancers [29, 37, 38]. As expected, knockdown of TMBIM6 suppressed NSCLC cell proliferation, migration, invasion and promoted cell apoptosis (Fig. 10a-c; Additional files Fig. S6). To evaluate whether TMBIM6 was required for the effect of STRIP2-IGF2BP3 on NSCLC progression, we knocked down TMBIM6 expression in PC9 cells, and then overexpressed STRIP2 or IGF2BP3 in these cells. Strikingly, TMBIM6 knockdown partly abrogated the promoting effects of STRIP2 overexpression and IGF2BP3 overexpression on cell proliferation, migration and invasion (Fig. 10d-e). In addition, the correlations between STRIP2 or IGF2BP3 and TMBIM6 were analyzed and showed that STRIP2 and IGF2BP3 expression levels were positively correlated with TMBIM6 level (Additional files Fig. S7). And immunohistochemistry analysis of 48 pairs NSCLC and their matched adjacent normal lung tissues indicated that increased STRIP2 expression was associated with high IGF2BP3 and TMBIM6 levels (Fig. 10f and g). Furthermore, NSCLC patients with high three signatures (STRIP2, IGF2BP3 and TMBIM6) expression manifested a shorter overall survival (*n* = 478, HR = 1.9, *P* = 4e-05) and disease-free survival (n = 478, HR = 1.5, *P* = 0.012) than patients with low three signatures expression (Fig. 10h). Overall, these results indicate that STRIP2 and IGF2BP3 promote NSCLC progression partially through TMBIM6, suggesting the STRIP2/IGF2BP3/TMBIM6 axis is critical to NSCLC pathogenesis and the prognosis of patients with NSCLC.

## Discussion

Metastasis is the major cause of death in NSCLC [39]. The dysregulated expression of metastasis-associated genes has been linked to the initiation, progression and prognosis of NSCLC [40]. In addition, STRIPAK complexes have shown to play an important role in cell growth, differentiation, proliferation and apoptosis of metabolism, immune regulation and tumor metastasi s[11]. However, the current understanding of the function and possible mechanism of STRIP2 (STRIPAK major component) in NSCLC tumorigenesis is lacking. Therefore, the present study aimed to investigate the biological role and molecular mechanism of STRIP2 in NSCLC. It was identified that STRIP2 was highly expressed in NSCLC tissues and cell lines, which was consistent with previous report [16]. Mechanistically, P300/CBP-mediated H3K27ac transcription in the promoter of STRIP2 induces STRIP2 transcription. In addition, it was also indicated that high STRIP2 expression was positively associated with positive lymph node metastasis, poor tumor differentiation, positive cancer thrombus and advanced stage of patients with NSCLC, suggesting that STRIP2 may play an oncogenic role in NSCLC tumorigenesis.

Accumulating evidence has demonstrated that the STRIPAK genes were aberrantly expressed and served as a prognostic value in various cancer, such as STRN4, [41] STRN3, [12] STRIP1, [12, 42] STRIP2, [12, 16] MST4, [43] MOB4, [43] SLMAP, [11] PPP2R1A, [11] PDCD10 [11] and CTTNBP2NL [11]. In the present study, survival analysis using Kaplan-Meier Plotter online datasets revealed that patients with high STRIP2 expression had a poorer prognosis compared with those with low STRIP2 expression, suggesting that STRIP2 could be considered as a prognostic marker for NSCLC patients.

It had been reported that STRIP2 has been shown to promote cell growth and migration in lung adenocarcinoma, [16] however, the molecular mechanism for the promotion of NSCLC progression remains enigmatic. Here, it was discovered that STRIP2 directly interacts with IGF2BP3 protein and cooperatively stabilizes the TMBIM6 in an m6A-dependent manner, potentially promoting NSCLC tumorigenesis. Increasing evidence has indicated that IGF2BP3 is highly expressed and promotes tumor progression in NSCLC, [44] however, the molecular mechanism of IGF2BP3 in NSCLC has not been elucidated. Here, we discovered that IGF2BP3 contributes to lung tumorigenesis dependent on STRIP2, which regulates common target TMBIM6. TMBIM6 is involved in tumor progression and metastasis in NSCLC [37, 45]. In addition, STRIP2 upregulated IGF2BP3 transcription and protein levels, thus leading to formulate a STRIP2-IGF2BP3 positive loop in NSCLC progression. IGF2BP3 was considered as an m6A reader and contributed to tumor progression through mediating the stabilization of IGF2BP3 targets in an m6A-dependent manner [35, 46, 47]. Our finding supports that STRIP2-IGF2BP3 axis stabilizes the TMBIM6 level in an m6A-dependent manner. However, whether STRIP2 involved in this process through m6A remains unclear.

TMBIM6, also named as transmembrane Bax inhibitor motif containing-6, is an inhibitor of Bax-induced apoptosis [48]. Growing evidences has indicated that TMBIM6 exert an oncogenic role in multiple cancers, including squamous cervical, non-small cell lung, breast, nasopharyngeal, hepatocellular, glioblastoma, and laryngeal squamous cell carcinoma [29, 37, 45, 49–53]. Our study implied that TMBIM6 was modified by m6A in the transcription level and interacts with IGF2BP3 and STRIP2, resulting in the stabilization of TMBIM6. In addition, knockdown of TMBIM6 suppressed cell proliferation, migration and invasion dependent on STRIP2 and IGF2BP3. Furthermore, TMBIM6 expression was positively correlated with STRIP2 and IGF2BP3 expression, and patients with high expression of the three gene signatures group show poorer overall survival and disease free survival than those patients with low expression of three gene signatures group, suggesting STRIP2-IGF2BP3-TMBIM6 axis has an important role in NSCLC progression and provides a novel potential prognostic biomarker for NSCLC.

## Conclusions

**Fig. 11:**
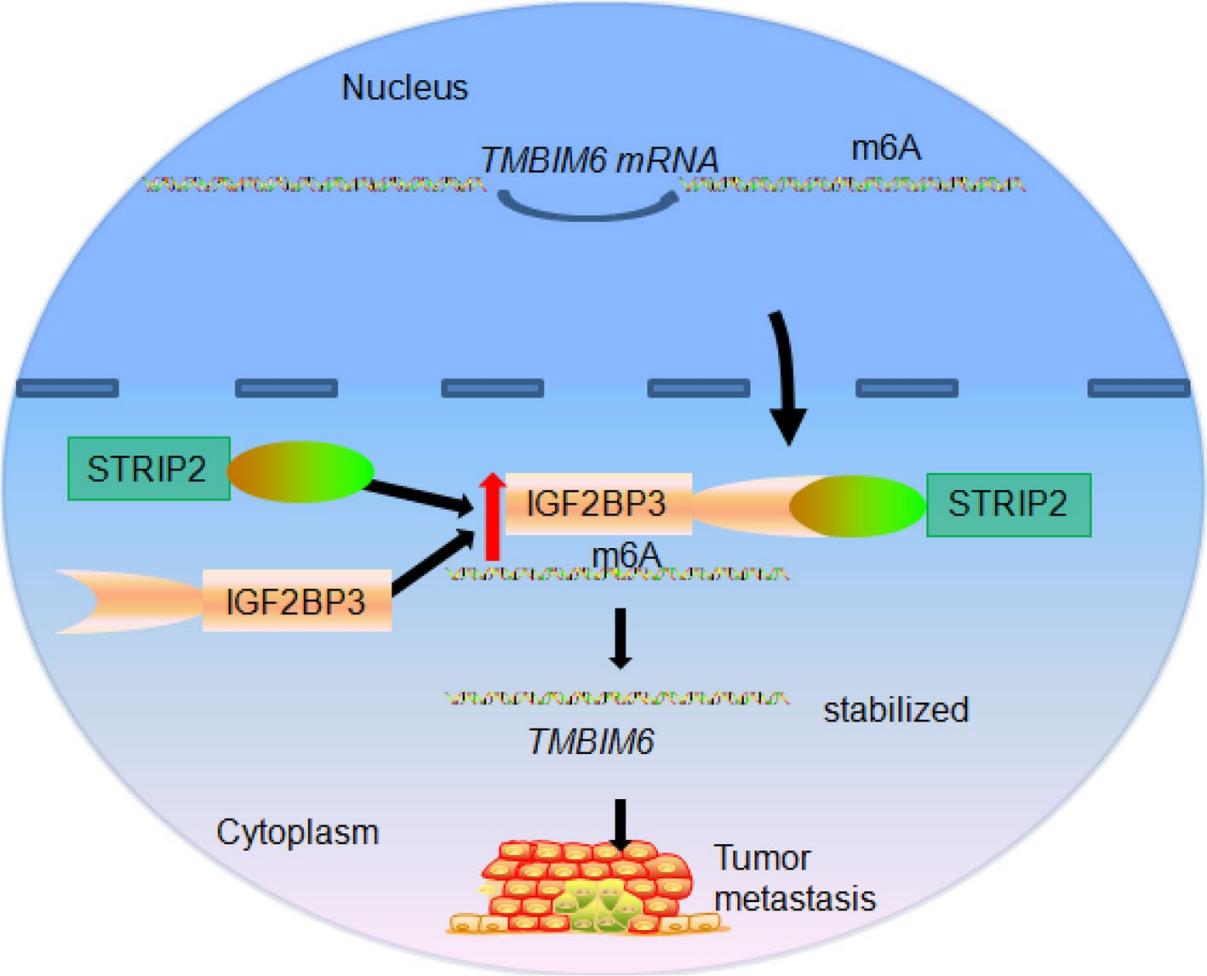
Graphic abstract of molecular mechanisms of STRIP2 promoting tumor progression in NSCLC

Overall, our study reveals that STRIP2 is an oncogene that mediates the stabilization of TMBIM6 level in an m6A-dependent manner in NSCLC (Fig. 11).

## Supplementary Information

**Additional file 1: Table S1.** Relationship between STRIP2 expression in NSCLC and clinicalpathological characteristics. **Table S2.** Sequence of primers used for qRT-PCR. **Table S3.** Genes of mass spectrometry. **Table S4.** Downregulated gene expression of STRIP2 knockdown RNA-sequence data. **Table S5.** 16 overlapping genes of STRIP2 knockdown RNA-sequence data and two published IGF2BP3 RIP-sequence data and GEO database (GSE90684). **Fig. S1.** The expression levels of P300 and CBP were increased in NSCLC. **Fig. S2.** C646 does not affect A549 and PC9 cell viabilities. **Fig. S3.** IGF2BP3 did not affect STRIP2 expression. **Fig. S4.** The number of different genes of STRIP2 knockdown RNA-sequence. **Fig. S5.** IGF2BP3 affected the content of m6A positive TMBIM6 level. **Fig. S6.** Knockdown of TMBIM6 promoted NSCLC cell apoptosis. **Fig. S7.** The correlations between STRIP2 or IGF2BP3 and TMBIM6.

## Data Availability

All datasets used and/or analyzed in the study are available from the corresponding authors on reasonable request.

## Abbreviations

NSCLC: Non-small cell lung cancer
FFPE: Formalin-Fixed Paraffin-Embedded
STRIPAK: Striatin-interacting phosphatase and kinase
STRIP2: Striatin interacting protein 2
IGF2BP3: Insulin-like growing factor II mRNA binding protein 3
TMBIM6: Transmembrane Bax inhibitor motif containing-6
H3K27ac: H3K27 acetylation
CHIP: Chromatin immunoprecipitation
MeRIP-qPCR: Methylated RNA immunoprecipitation-PCR
