# Supplementary material for: STRIP2 motivates non-small cell lung cancer progression by modulating the TMBIM6 stability through IGF2BP3 dependent
Source: J Exp Clin Cancer Res. 2023 Jan 13;42:19. doi: 10.1186/s13046-022-02573-1 (PMC9837939; doi:10.1186/s13046-022-02573-1)
Supplement: Supplementary file 1 — Additional file 1: Table S1. Relationship between STRIP2 expression in NSCLC and clinicalpathological characteristics. Table S2. Sequence of primers used for qRT-PCR. Table S3. Genes of mass spectrometry. Table S4. Downregulated gene expression of STRIP2 knockdown RNA-sequence data. Table S5. 16 overlapping genes of STRIP2 knockdown RNA-sequence data and two published IGF2BP3 RIP-sequence data and GEO database (GSE90684). Fig. S1. The expression levels of P300 and CBP were increased in NSCLC. Fig. S2. C646 does not affect A549 and PC9 cell viabilities. Fig. S3. IGF2BP3 did not affect STRIP2 expression. Fig. S4. The number of different genes of STRIP2 knockdown RNA-sequence. Fig. S5. IGF2BP3 affected the content of m6A positive TMBIM6 level. Fig. S6. Knockdown of TMBIM6 promoted NSCLC cell apoptosis. Fig. S7. The correlations between STRIP2 or IGF2BP3 and TMBIM6. [file 13046_2022_2573_MOESM1_ESM.docx]

**Additional files**

**Supplementary Tables**

**Table S1. Relationship between STRIP2 expression in NSCLC and clinicalpathological characteristics**

| Variables | Low expression group (n=51) | High expression  group (n=138) | χ^2^ | *p* |
| --- | --- | --- | --- | --- |
| Gender |  |  | 0.178 | 0.673 |
| Female | 23 | 67 |  |  |
| Male | 28 | 71 |  |  |
| Age |  |  | 0.007 | 0.934 |
| >65 | 24 | 64 |  |  |
| ≤65 | 27 | 74 |  |  |
| Smoking history |  |  | 0.014 | 0.905 |
| Ever | 26 | 69 |  |  |
| Never | 25 | 69 |  |  |
| Tumor size (cm) |  |  | 0.043 | 0.836 |
| >3.0 | 14 | 40 |  |  |
| ≤3.0 | 37 | 98 |  |  |
| Tumor differentiation |  |  | 2.984 | 0.041* |
| Well/Moderate | 43 | 103 |  |  |
| Poor | 8 | 35 |  |  |
| Lymphatic invasion |  |  | 3.928 | 0.026* |
| Present | 14 | 52 |  |  |
| Absent | 37 | 86 |  |  |
| Cancer thrombus |  |  | 3.166 | 0.045* |
| Present | 16 | 62 |  |  |
| Absent | 35 | 76 |  |  |
| Stage |  |  | 3.711 | 0.038* |
| I+II | 36 | 79 |  |  |
| III+ IV | 15 | 59 |  |  |

**Table S2. Sequence of primers used for** **qRT-PCR**

| **Genes** | **Primers** |
| --- | --- |
| STRIP2-forward | 5’-AGGTGGTCAGTAGGGAACGG-3’ |
| STRIP2-reverse | 5’-TGTAGCACATCGACCTCTGAA-3’ |
| IGF2BP3-forward | 5’-TATATCGGAAACCTCAGCGAGA-3’ |
| IGF2BP3-reverse | 5’-GGACCGAGTGCTCAACTTCT-3’ |
| TMBIM6-forward | 5’-CATATAACCCCGTCAACGCAG-3’ |
| TMBIM6-reverse | 5’-GCAGCCGCCACAAACATAC-3’ |
| TSPAN17-forward | 5’-GCTGCTGCGGGAAATACTTC-3’ |
| TSPAN17-reverse | 5’-AGAGGCCGATAGCCAGGAA-3’ |
| KREMEN1-forward | 5’-CCCGAGTGTTTCACAGCCAAT-3’ |
| KREMEN1-reverse | 5’-GGATGCTGGAAAGTCTCGTTC-3’ |
| BCL3-forward | 5’-CCGGAGGCGCTTTACTACC-3’ |
| BCL3-reverse | 5’-TAGGGGTGTAGGCAGGTTCAC-3’ |
| OTUD6B-forward | 5’-TGAGAAGGCATCGCAAAGAGA-3’ |
| OTUD6B-reverse | 5’-ATCTTCGGTGAGTTGCTTCCT-3’ |
| CDK2AP1-forward | 5’-ATGTCTTACAAACCGAACTTGGC-3’ |
| CDK2AP1-reverse | 5’-GCCCGTAGTCACTGAGCAG-3’ |
| ABHD14B-forward | 5’-TCACTGAGTGGCATGTACTCC-3’ |
| ABHD14B-reverse | 5’-GTTGGCAGCATTGATTTTGTCA-3’ |
| EIF5-forward | 5’-AGCGTGTCAGACCAGTTCTAT -3’ |
| EIF5-reverse | 5’-CTGTCTTGATTCCATTGCCTTTG-3’ |
| c-MYC-forward | 5’-TTCGGGTAGTGGAAAACCAG -3’ |
| c-MYC-reverse | 5’-AGTAGAAATACGGCTGCACC -3’ |
| P300-forward | 5’-GCTTCAGACAAGTCTTGGCAT-3’ |
| P300-reverse | 5’-ACTACCAGATCGCAGCAATTC-3’ |
| CBP-forward | 5’-CGGCTCTAGTATCAACCCAGG-3’ |
| CBP-reverse | 5’-TTTTGTGCTTGCGGATTCAGT-3’ |
| 18sRNA-forward | 5’-GTAACCCGTTGAACCCCATT-3’ |
| 18sRNA-reverse | 5’-CCATCCAATCGGTAGTAGCG-3’ |

**Table S3. Genes of mass spectrometry**

| Gene | Unique Sequence | M.W. (Da) | MS Score |
| --- | --- | --- | --- |
| IGF2BP3 | 11 | 63704.6 | 278 |
| IGF2BP2 | 3 | 66121 | 184 |
| STAU1 | 3 | 63181.9 | 142 |
| RBM14 | 3 | 69490.9 | 62.65 |
| CMC2 | 3 | 74174.9 | 42.8 |
| AINX | 3 | 55389.9 | 39.3 |
| HP1B3 | 2 | 61206.5 | 39.2 |
| HNRPQ | 2 | 69602.3 | 38.9 |
| PPBN | 4 | 57376.5 | 31.3 |
| DDX5 | 4 | 69147.6 | 29.2 |
| SERA | 3 | 56650 | 28.4 |
| GNL3 | 2 | 61992.7 | 21.6 |
| HSP7C | 13 | 70897.6 | 19.47 |
| NONO | 2 | 54231.3 | 19.3 |
| G3BP2 | 2 | 54120.9 | 18.6 |
| EZRI | 1 | 69412.3 | 17.7 |
| TCPZ | 1 | 58023.6 | 17.2 |
| G6PD | 1 | 59256.3 | 16.2 |
| FXR1 | 7 | 69720.3 | 11.83 |
| DDX3X | 4 | 73242.8 | 10.73 |
| PABP4 | 4 | 70782.3 | 10.27 |
| LMNA | 3 | 74138.8 | 8.45 |
| FXR2 | 4 | 74222.7 | 6.59 |
| PTBP1 | 3 | 57220.9 | 6.03 |
| GRP75 | 5 | 73679.9 | 5.99 |
| PABP1 | 4 | 70670.4 | 5.89 |
| ATPB | 5 | 56559.4 | 5.41 |
| IMDH2 | 4 | 55804.5 | 5.17 |

**Table S4. Downregulated gene expression of STRIP2 knockdown RNA-sequence data**

| Number | Gene name | Number | Gene name | Number | Gene name |
| --- | --- | --- | --- | --- | --- |
| 1 | AL136295 | 86 | BMF | 171 | AEN |
| 2 | ISY1-RAB43 | 87 | WNT4 | 172 | SLC26A2 |
| 3 | AC234781 | 88 | AL109615 | 173 | TMEM201 |
| 4 | AC008763 | 89 | AC010733 | 174 | MGAM |
| 5 | AC016747 | 90 | KREMEN1 | 175 | ABHD14B |
| 6 | AP003071 | 91 | CSPG4 | 176 | RTKN2 |
| 7 | MIR1244-1 | 92 | ANKRD36C | 177 | SULT1A3 |
| 8 | AC008878 | 93 | PLIN4 | 178 | CDK2AP1 |
| 9 | CLDN4 | 94 | CHD5 | 179 | PALM2AKAP2 |
| 10 | AC244197 | 95 | STRIP2 | 180 | EIF5 |
| 11 | CCL20 | 96 | ZNF133 | 181 | CHAC2 |
| 12 | UBE2F-SCLY | 97 | PDK4 | 182 | FAM216A |
| 13 | NPIPP1 | 98 | MICAL2 | 183 | NCLN |
| 14 | AL121594 | 99 | AL109615 | 184 | F11R |
| 15 | AC009133 | 100 | DUBR | 185 | SERPINB7 |
| 16 | EGR2 | 101 | TREML4 | 186 | LINC01836 |
| 17 | AC015712 | 102 | AL732372 | 187 | UBE2D4 |
| 18 | CEP170P1 | 103 | SRRM3 | 188 | NBPF14 |
| 19 | LRRC32 | 104 | ALDH1A1 | 189 | AL135905 |
| 20 | NDUFC2-KCTD14 | 105 | CXCL1 | 190 | MPZL1 |
| 21 | IQCN | 106 | CA2 | 191 | HTR7 |
| 22 | ZNF843 | 107 | TSN | 192 | SGSH |
| 23 | HERC2P7 | 108 | RHCG | 193 | TMEM198 |
| 24 | SHLD2P1 | 109 | MRPS9 | 194 | MTAP |
| 25 | TRAPPC5 | 110 | ANXA2R | 195 | OTUD6B |
| 26 | MAP1A | 111 | MOGS | 196 | ENTPD1 |
| 27 | IL1A | 112 | PDP2 | 197 | SOCS2 |
| 28 | TRIM31 | 113 | SPRR1B | 198 | SUSD2 |
| 29 | GRIN3B | 114 | NR1D1 | 199 | MRPL20-AS1 |
| 30 | G0S2 | 115 | SPRR1A | 200 | GABBR2 |
| 31 | FBN2 | 116 | PDZK1IP1 | 201 | GRB2 |
| 32 | LTB | 117 | SIRPG | 202 | NABP2 |
| 33 | S100A8 | 118 | CYP2T1P | 203 | RN7SL1 |
| 34 | AL121761 | 119 | TNS1 | 204 | AL162595 |
| 35 | AP002990 | 120 | SENP3-EIF4A1 | 205 | VGF |
| 36 | TH | 121 | BCL2L11 | 206 | DIO2 |
| 37 | IL1B | 122 | CAPN5 | 207 | DDX20 |
| 38 | TOMM6 | 123 | KCTD18 | 208 | RAPH1 |
| 39 | TOLLIP-AS1 | 124 | SLC30A3 | 209 | PSG5 |
| 40 | CES1 | 125 | STARD4 | 210 | EPGN |
| 41 | LINC02029 | 126 | VIM | 211 | FSTL4 |
| 42 | ADORA2A | 127 | PER1 | 212 | GALNT4 |
| 43 | AC009812 | 128 | SGPP2 | 213 | SIX2 |
| 44 | PDXDC2P | 129 | WDR6 | 214 | NEXN |
| 45 | RDH16 | 130 | SLC28A3 | 215 | SNX27 |
| 46 | ATP6V1G2-DDX39B | 131 | GABARAPL1 | 216 | GJA5 |
| 47 | CORO2B | 132 | SAMD11 | 217 | CCDC137 |
| 48 | SGK2 | 133 | PNRC1 | 218 | SCARA3 |
| 49 | PTX3 | 134 | PECAM1 | 219 | Z92544 |
| 50 | SLX1A-SULT1A3 | 135 | S100A9 | 220 | MYOZ1 |
| 51 | ANXA10 | 136 | AL162151 | 221 | C5orf24 |
| 52 | AC073508 | 137 | PADI4 | 222 | FAM83H |
| 53 | AC010323 | 138 | POLI | 223 | PEG10 |
| 54 | KRT6B | 139 | TSPAN17 | 224 | ZNF778 |
| 55 | RGCC | 140 | ARL14 | 225 | PRR5L |
| 56 | GCNT3 | 141 | CLIP2 | 226 | C4BPB |
| 57 | CDKN1C | 142 | AC024560 | 227 | DKK1 |
| 58 | AC027644 | 143 | LINC00514 | 228 | BDNF |
| 59 | BDKRB1 | 144 | GBP2 | 229 | ANKRD1 |
| 60 | RALB | 145 | BTN3A2 | 230 | SLC15A2 |
| 61 | AC004997 | 146 | ST6GALNAC2 | 231 | CPA4 |
| 62 | ANPEP | 147 | HDAC9 | 232 | GREM1 |
| 63 | LINC00887 | 148 | DUSP6 | 233 | ZNF786 |
| 64 | MME | 149 | PEX2 | 234 | TLCD4-RWDD3 |
| 65 | SOD2 | 150 | NR5A2 | 235 | RN7SL2 |
| 66 | AP000696 | 151 | FAM167A | 236 | ADAT3 |
| 67 | TIAF1 | 152 | CDON | 237 | AC092032 |
| 68 | AC012181 | 153 | TMC8 | 238 | NKX1-2 |
| 69 | SCNN1G | 154 | ZNF862 | 239 | HLX |
| 70 | BX537318 | 155 | ALDOC | 240 | PVRIG2P |
| 71 | TNF | 156 | DOCK4 | 241 | ZNF8-ERVK3-1 |
| 72 | AC015660 | 157 | TMBIM6 | 242 | MRPS31P5 |
| 73 | RGS17 | 158 | WFDC2 | 243 | AC004593 |
| 74 | ZNF467 | 159 | AKR1C1 | 244 | IGFBP5 |
| 75 | RGS2 | 160 | JAK3 | 245 | AC004922 |
| 76 | TFAP2E | 161 | SPATA2 | 246 | KRT4 |
| 77 | AC006064 | 162 | AOC1 | 247 | AC023055 |
| 78 | BDKRB2 | 163 | ATXN7 | 248 | C8orf44-SGK3 |
| 79 | KLF4 | 164 | BCL3 | 249 | ARPIN-AP3S2 |
| 80 | ZNF490 | 165 | GPANK1 | 250 | BCL2L2-PABPN1 |
| 81 | BTBD3 | 166 | DUSP13 | 251 | AL133355 |
| 82 | NUDT16 | 167 | LNX1 | 252 | AC004069 |
| 83 | ZMIZ1-AS1 | 168 | TNFAIP3 | 253 | FP565260 |
| 84 | DEFB1 | 169 | MTCO1P12 | 254 | AC117378 |
| 85 | HEY1 | 170 | SPIN1 | 255 | MIR4707 |

**Table S5. 16 overlapping genes of STRIP2 knockdown RNA-sequence data and two published IGF2BP3 RIP-sequence data and GEO database (GSE90684).**

| Number | Gene name |
| --- | --- |
| 1 | CDK2AP1 |
| 2 | EIF5 |
| 3 | BTBD3 |
| 4 | ZNF133 |
| 5 | WDR6 |
| 6 | ATXN7 |
| 7 | ABHD14B |
| 8 | STARD4 |
| 9 | KREMEN1 |
| 10 | GABARAPL1 |
| 11 | BCL3 |
| 12 | TSN |
| 13 | TSPAN17 |
| 14 | MOGS |
| 15 | OTUD6B |
| 16 | TMBIM6 |

**Supplementary Figures**

**Fig. S1: The expression levels of P300 and CBP were increased in NSCLC.**


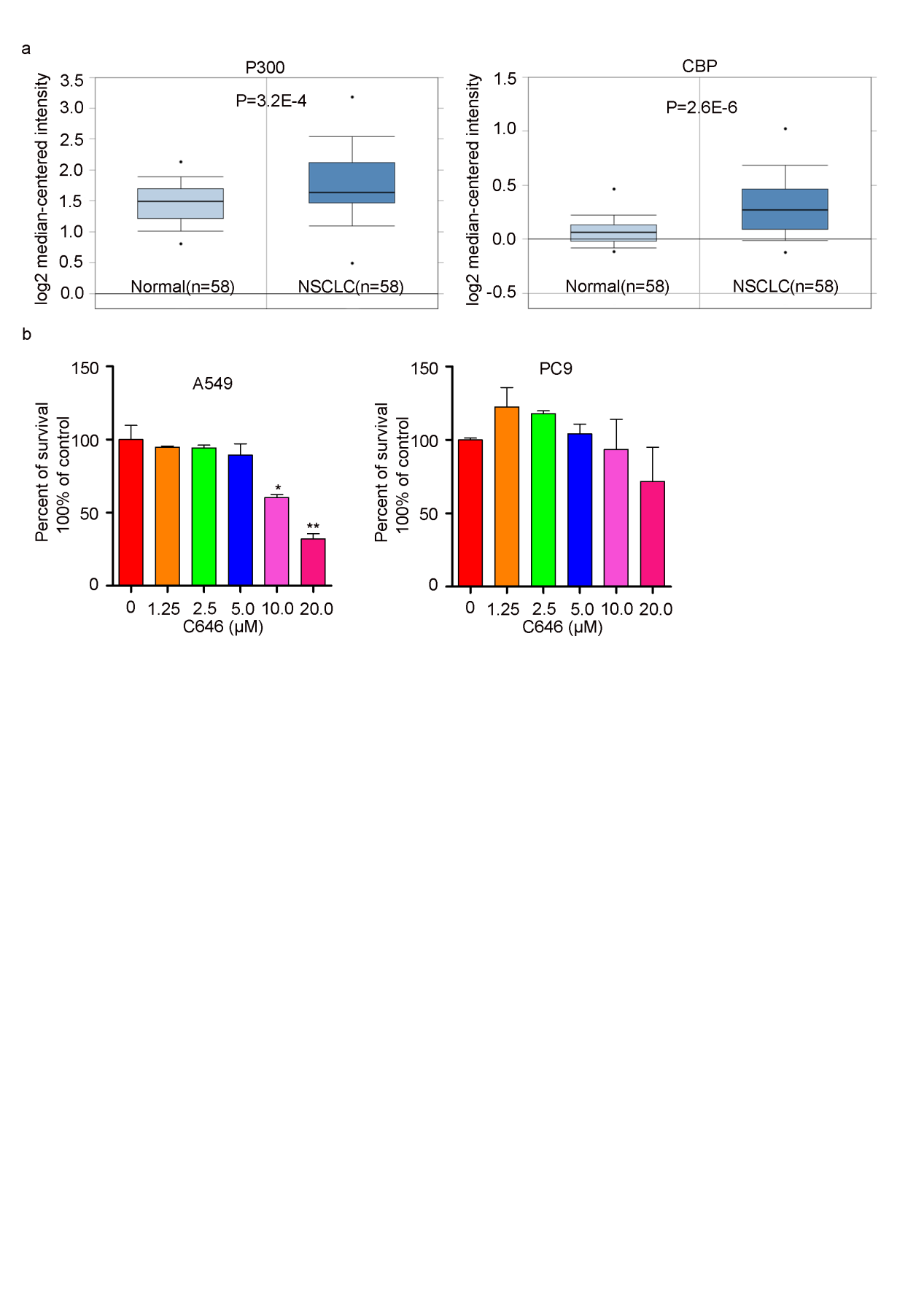


P300 and CBP mRNA levels were elevated in GEO database GSE32863.

**Fig. S2: C646 does not affect A549 and PC9 cell viabilities.**


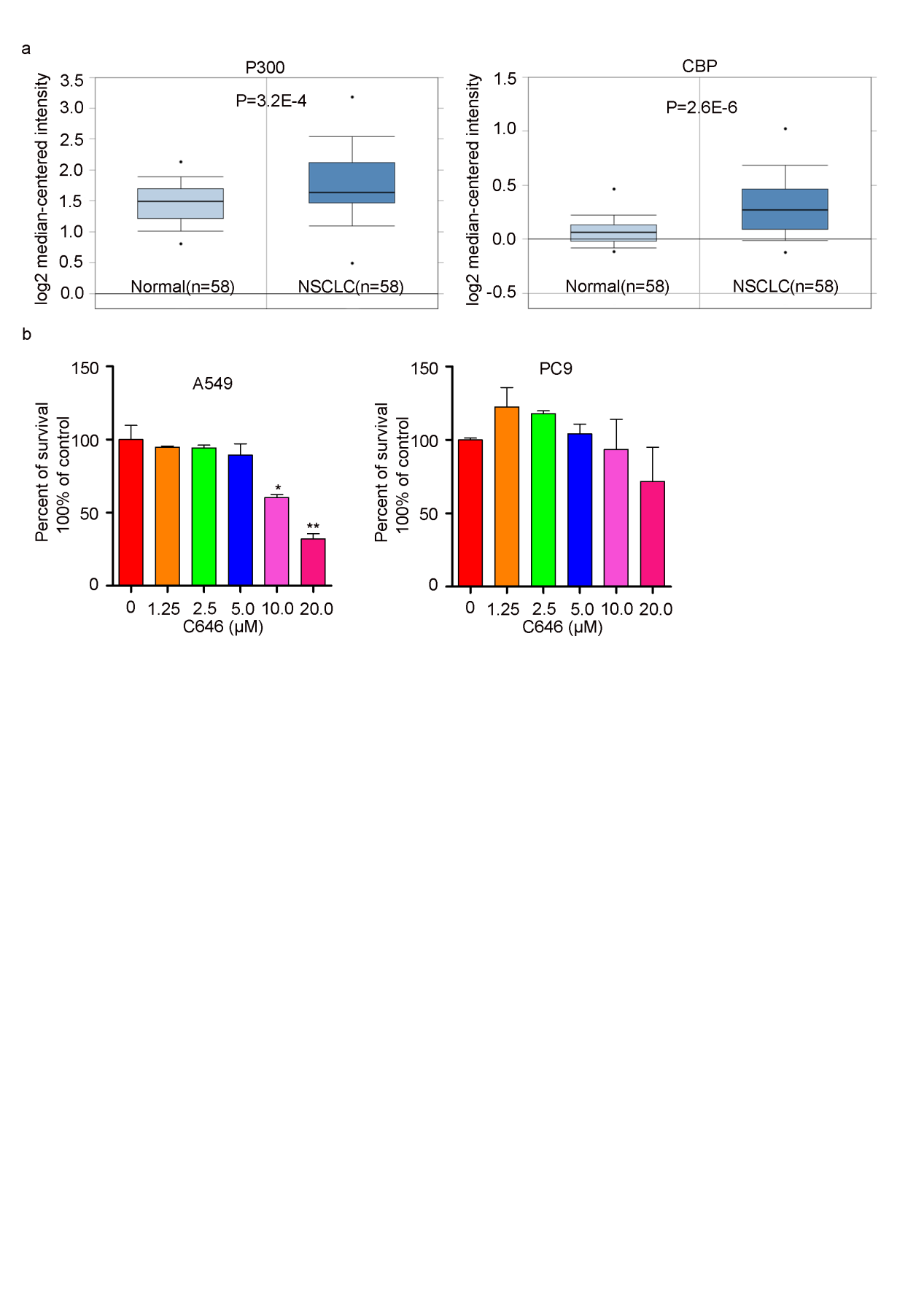


Cells were treated with different concentrations of C646 for 24 hours, and cell viabilities were measured using CCK-8 assay.

**Fig. S3: IGF2BP3 did not affect STRIP2 expression.**


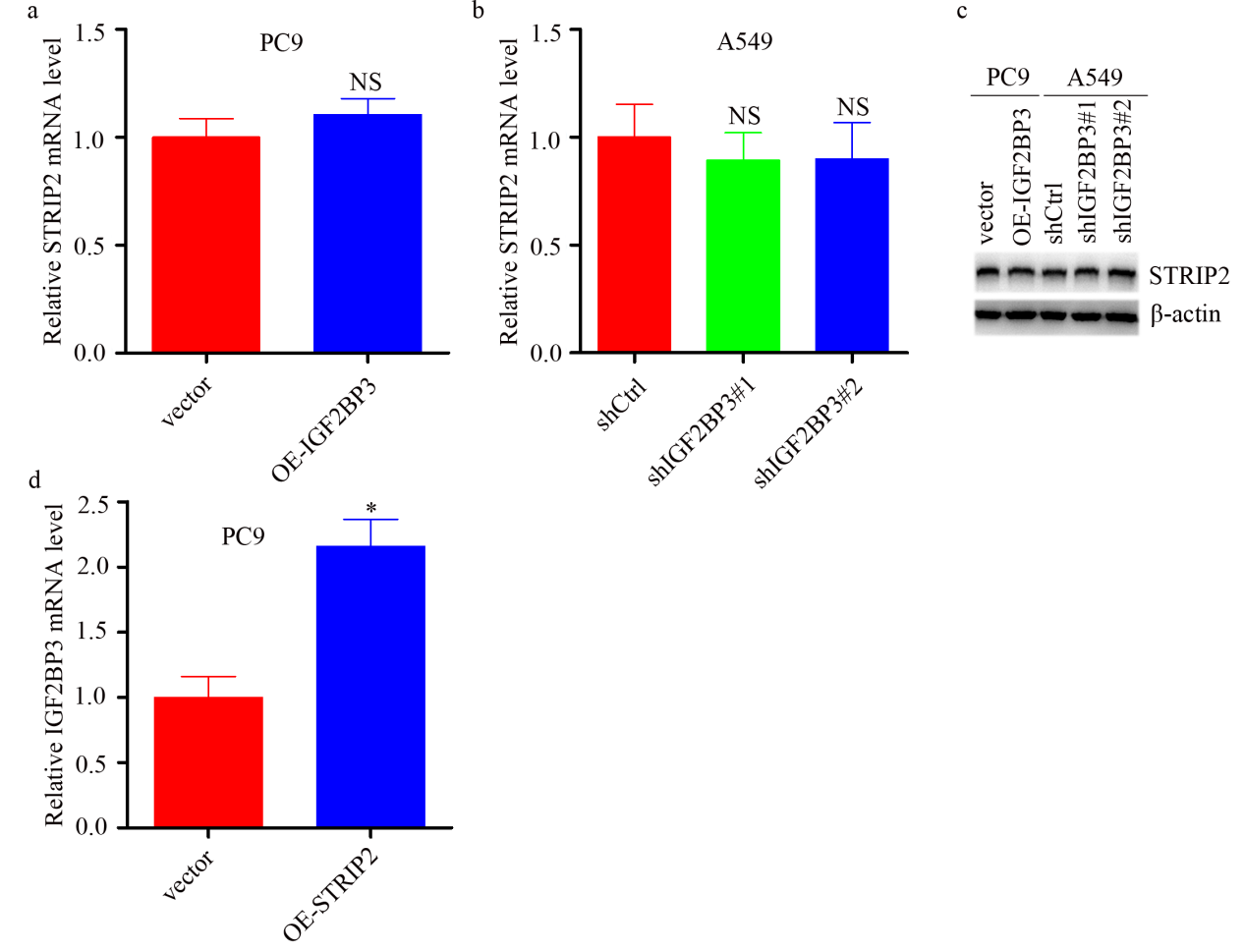


a: The mRNA expression level of STRIP2 was detected using qRT-PCR in PC9 cells transfected with vector and IGF2BP3 plasmids; b: The mRNA expression level of STRIP2 was detected using qRT-PCR in A549 cells; c: STRIP2 protein level was measured using western blotting in PC9 and A549 cells; d: The mRNA expression level of IGF2BP3 was detected using qRT-PCR in PC9 cells transfected with vector and STRIP2 plasmids. NS, not signifance; OE, overexpression.

**Fig. S4: The number of different genes of STRIP2 knockdown RNA-sequence.**


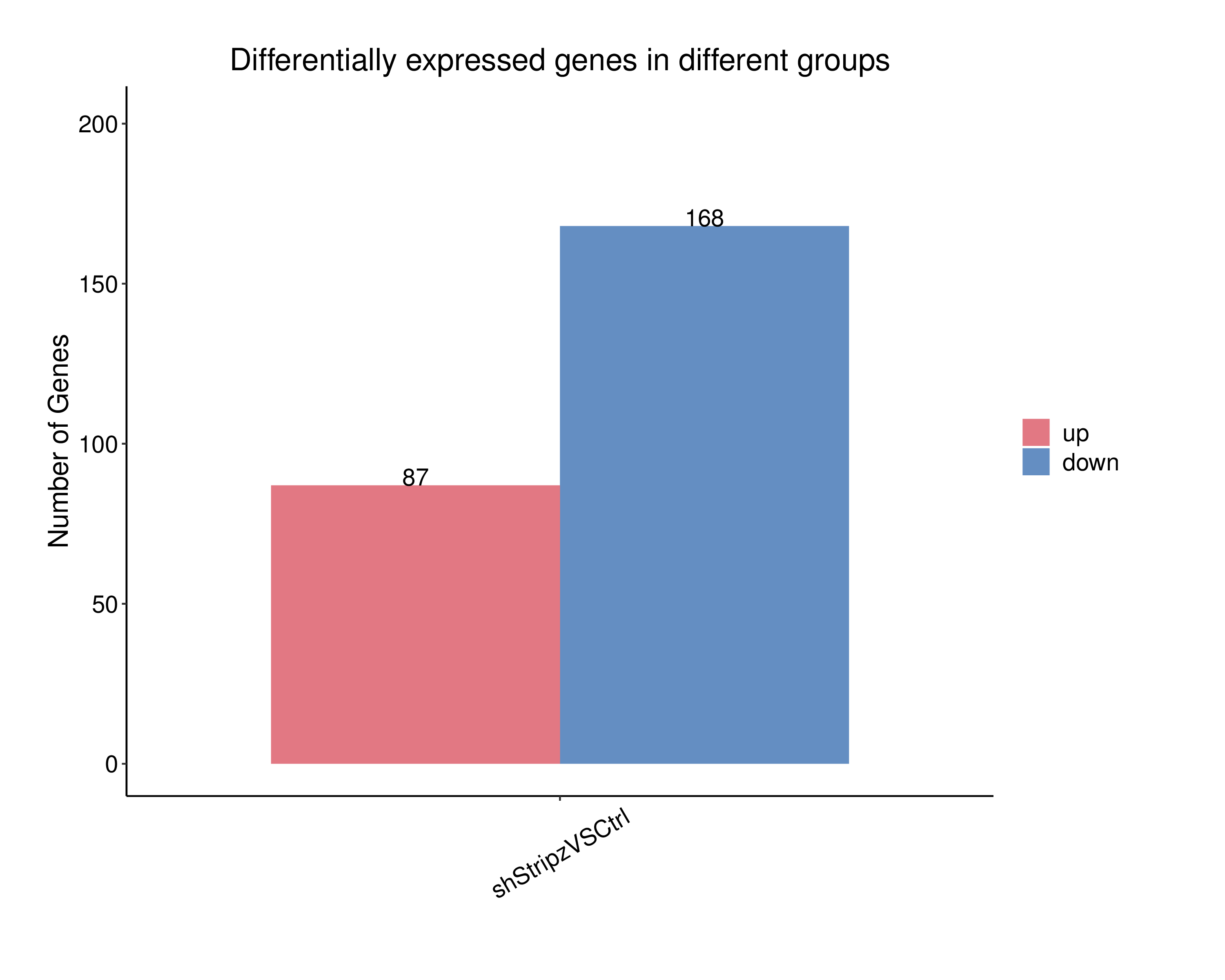


Abnormally expressed genes were analyzed in STRIP2 knockdown group compared with scramble control group.

**Fig. S5: IGF2BP3 affected the content of m6A positive TMBIM6 level.**

**
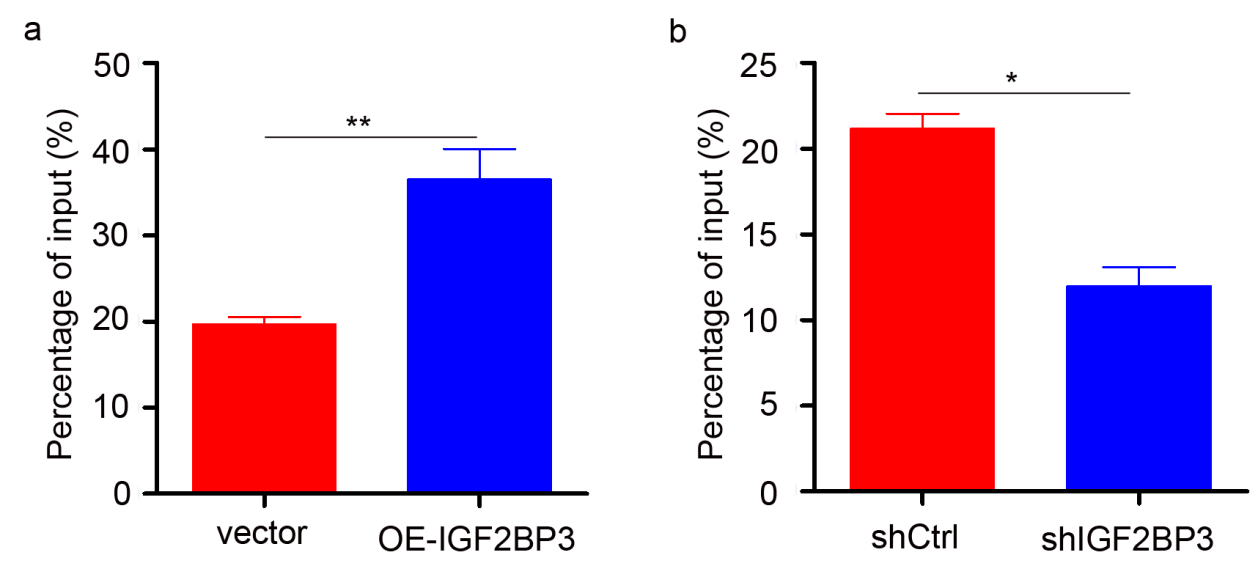
**

a: RIP-PCR analysis detecting the enrichment of TMBIM6 m6A modifications on overexpression of IGF2BP3; b: RIP-PCR analysis detecting the enrichment of TMBIM6 m6A modifications on knockdown of IGF2BP3. OE, overexpression.

**Fig. S6: Knockdown of TMBIM6 promoted NSCLC cell apoptosis.**

**
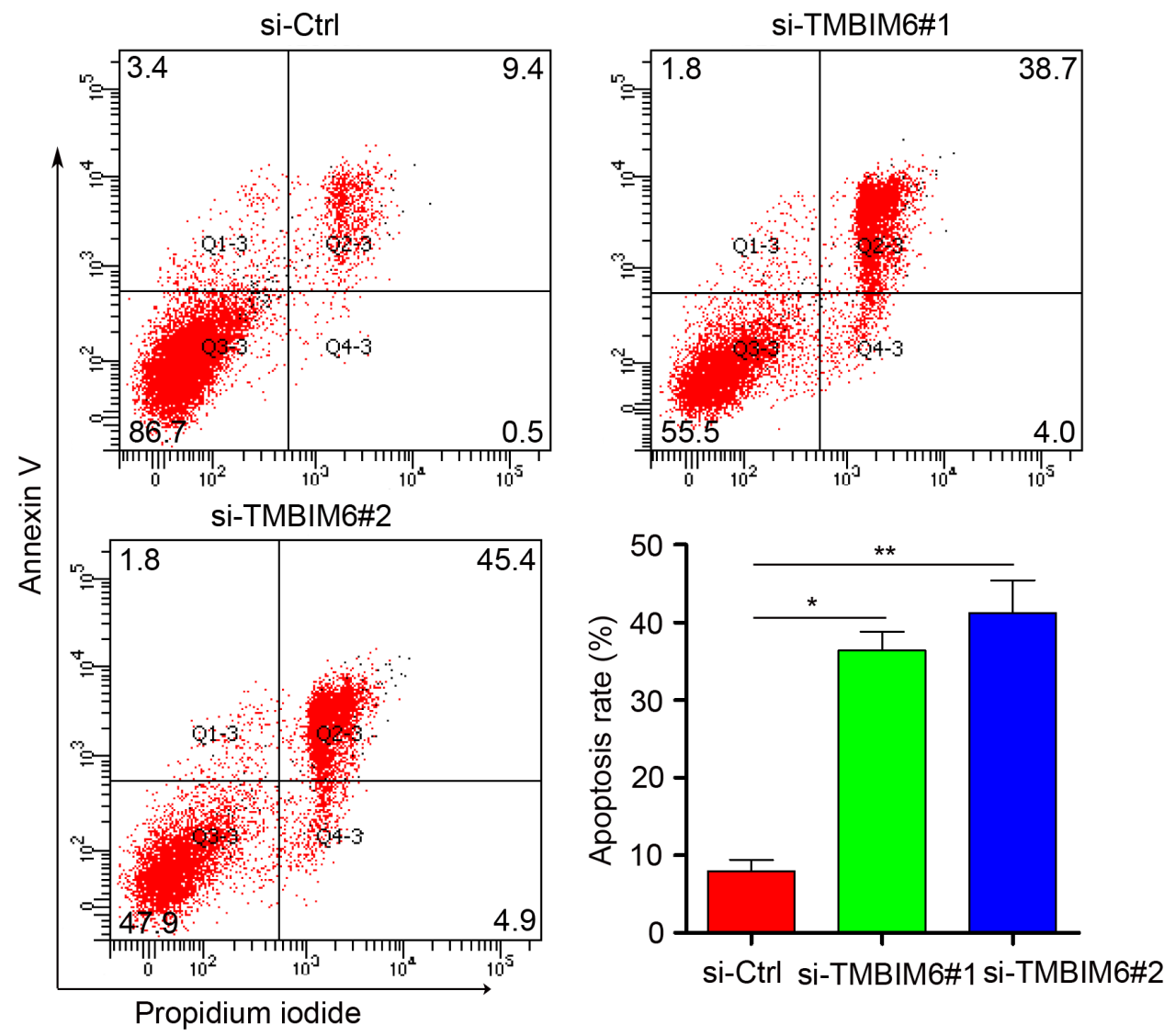
**

PC9 cells were transfected with two specific small interfering (si)RNAs of TMBIM6 and scrambled siRNA and then cell apoptosis were analyzed using FACS.

**Fig. S7: The correlations between STRIP2 or IGF2BP3 and TMBIM6.**


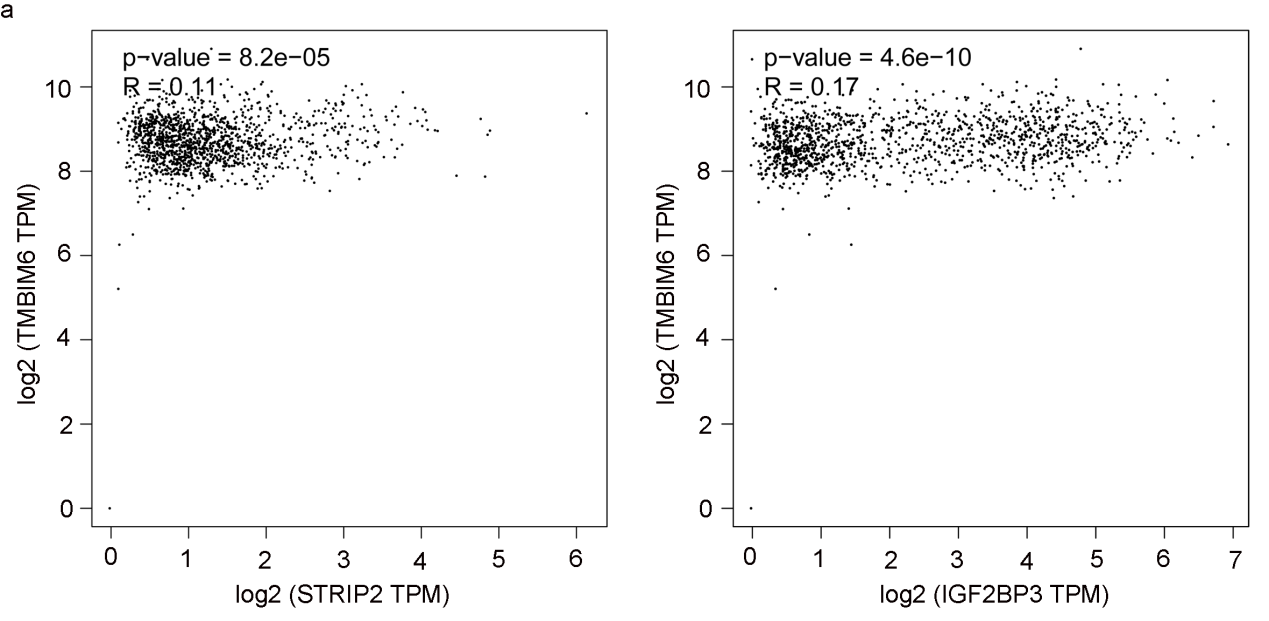


The data of STRIP2, IGF2BP3 and TMBIM6 expression levels were collected from GEPIA2 (http://gepia2.cancer-pku.cn) and the correlations between STRIP2 or IGF2BP3 and TMBIM6 were analyzed.
